# Efficacy and safety of a novel pain management device, AT-04, for endometriosis-related pain: study protocol for a phase III randomized controlled trial

**DOI:** 10.1186/s12978-024-01739-8

**Published:** 2024-01-26

**Authors:** Hiroshi Ishikawa, Osamu Yoshino, Fuminori Taniguchi, Tasuku Harada, Mikio Momoeda, Yutaka Osuga, Tamiki Hikake, Youko Hattori, Michiko Hanawa, Yosuke Inaba, Hideki Hanaoka, Kaori Koga

**Affiliations:** 1 Department of Obstetrics and Gynecology, Graduate School of Medicine, Chiba University, Inohana 1-8-1, Chuo-Ku, Chiba, 260-8670 Japan; 2 Department of Obstetrics and Gynecology, Chiba University Hospital, Chiba, 260-8677 Japan; 3 Department of Obstetrics and Gynecology, University of Yamanashi Graduate School of Medicine, Yamanashi, 409-3898 Japan; 4 Department of Obstetrics and Gynecology, Faculty of Medicine, Tottori University, Tottori, 683-8504 Japan; 5 Department of Obstetrics and Gynecology, Aiiku Hospital, Tokyo, 105-8321 Japan; 6 Department of Obstetrics and Gynecology, The University of Tokyo Graduate School of Medicine, Tokyo, 113-8655 Japan; 7 Chiba University Clinical Research Center, Chiba University Hospital, Chiba, 260-8677 Japan; 8 Data Center, Chiba University Hospital, Chiba, 260-8677 Japan

**Keywords:** Endometriosis, Chronic pain, Quality of life, Pain management, Electromagnetic radiation, Patient reported outcome measures

## Abstract

**Background:**

Endometriosis-related pain encompassing dysmenorrhea, dyspareunia, and chronic pelvic pain, reduces the quality of life in premenopausal women. Although treatment options for endometriosis alleviate this pain, approximately one-third of women still experience pain even after receiving treatment, indicating the need for novel approaches to pain relief in those women. The Angel Touch device (AT-04) is a portable magnetic fields irradiation device that incorporates a combination of mixed alternative magnetic fields at 2 kHz and 83.3 MHz. A phase III trial confirmed the efficacy and safety of AT-02, a prototype of AT-04, for pain relief in patients with fibromyalgia.

**Methods:**

This is a phase III, multicenter, prospective, randomized, sham device-controlled, double-blind, parallel study. The participants will be premenopausal women aged > 18 years who have endometriosis-related pain with at least moderate severity. Considering dropouts, 50 participants have been deemed appropriate. Eligible women will be centrally registered, and the data center will randomly allocate them in a 1:1 ratio to the intervention and control groups. Women in the intervention group will receive electromagnetic wave irradiation generated by AT-04 and those who in the control group will wear a sham device for 16 weeks, and both groups will wear AT-04 for another 4 weeks. The primary outcome measure is the change in the Numeric Rating Scale score at 16 weeks compared with the baseline. Secondary outcome measures are efficacy for pelvic pain including dysmenorrhea and non-menstrual pain, and chronic pelvic pain not related to menstruation, dysmenorrhea, and dyspareunia, and improvement of quality of life during the study period. Safety will be evaluated by device defects and the frequency of adverse events. The study protocol has been approved by the Clinical Study Review Board of Chiba University Hospital, Chiba, Japan, and will be conducted in accordance with the principles of the Declaration of Helsinki and the Japanese Clinical Trials Act and relevant notifications.

**Discussion:**

This study aims to develop a novel method of managing endometriosis-related pain. The AT-04 is an ultralow-invasive device that can be used without inhibiting ovulation, suggesting potential benefits to women of reproductive-age.

*Trial registration number* Japan Registry of Clinical Trials (jRCTs032230278).

**Supplementary Information:**

The online version contains supplementary material available at 10.1186/s12978-024-01739-8.

## Plain language summary

Endometriosis is a chronic inflammatory disorder that negatively impacts reproductive health via endometriosis-related pain, infertility, and endometriosis-associated ovarian cancer. Although current therapeutic options for endometriosis are effective for the endometriosis-related pain, approximately one-third of women still experience pain even after receiving treatment, indicating the need for novel approaches to pain relief in those women. This is the first randomized controlled trial to investigate the efficacy and safety of a novel portable pain management device, AT-04, that incorporates a combination of mixed alternating magnetic fields, for endometriosis-related pain. This is a multicenter, prospective, sham device-controlled, double-blind, parallel study. Enrolled women will have undergone standard hormonal treatment for endometriosis at baseline, and this allows for assessing whether the device remains effective when used in conjunction with existing treatment methods. The study also will explore the impact of AT-04 on reducing the size of ovarian endometriotic cysts that reflect the activity of endometriosis. The study reflects the strong desire by physicians to liberate women from the unbearable pain associated with endometriosis. The sole efficacy of AT-04 in treating endometriosis-related pain is difficult to evaluate as there is a possibility that menstrual cycles may influence the assessment of pain and quality of life. However, the study findings regarding the effectiveness of AT-04 for the treatment of endometriosis-related pain may benefit women with endometriosis who have pain that is not effectively relieved by other treatments. Consequently, it may contribute to the improvement of reproductive health within society.

**Supplementary Information:**

The online version contains supplementary material available at 10.1186/s12978-024-01739-8.

## Background

Endometriosis is a progressive, chronic inflammatory disorder that affects approximately 10% of women of reproductive age. It generally develops in late adolescence to early adulthood, and the associated symptoms often appear in the late twenties and early thirties [1]. Women with endometriosis experience endometriosis-related pain, encompassing dysmenorrhea, dyspareunia, and chronic pelvic pain, which reduces their quality of life and decreases labor productivity. Although endometriosis-related infertility and ovarian malignant tumors arising from ovarian endometriotic cysts are also important, controlling endometriosis-related pain is of utmost importance when treating endometriosis [2]. Current standard treatments for endometriosis include progestin-based hormonal treatments, such as combined oral contraceptive (OC) pills, gonadotropin-releasing hormone (GnRH) analogs, and laparoscopic surgeries [3]. Analgesics and traditional Chinese medicines are also prescribed to relieve endometriosis-related pain; however, these treatments may be completely ineffective or insufficient in some women, even if appropriate treatments are administered [4]. Additionally, endometriosis-related pain often persists even after laparoscopic excision of endometriotic lesions, indicating that controlling disease progression does not necessarily lead to pain relief. Worsening pain symptoms can trigger anxiety and depression, leading to a further decrease in quality of life and labor productivity [5].

Endometriosis severity does not necessarily correlate with pain intensity, indicating that pain development in patients with endometriosis involves complex mechanisms. Hence, in addition to lowering the pain threshold due to persistent chronic pelvic pain, chronic inflammation, peripheral and central pain generators, endocrine changes, and structural alterations in the peripheral and central nervous systems are also associated with pain [6]. Upregulation of vascular endothelial factors and nerve growth factors as well as proliferation of sensory nerves in local endometriotic lesions may also be associated with pain [7]. Furthermore, the presence of treatment-resistant endometriosis-related pain may indicate the progression of local endometriotic lesions.

The Angel Touch device (AT-04, ait^®^, developed by Peace of Mind Co., Ltd., Kumamoto, Japan) is a portable magnetic fields irradiation device that incorporates a combination of mixed alternative magnetic fields at 2 kHz and 83.3 MHz (magnetic field energy is approximately one-third of the Earth’s magnetic field). Local pain relief may be achieved by carefully using this device on the affected area. Animal experimental data has revealed that the pain control mechanism of this device involves the regulation of nerve growth factors, local inhibition of inflammatory cytokines, and activation of the descending inhibitory system [8]. These are the possible mechanisms that control endometriosis-related pain. A phase III clinical trial confirmed the efficacy and safety of AT-02, a prototype of AT-04, for pain relief in patients with fibromyalgia [9]. Moreover, a pilot study using AT-04 for pain relief from dysmenorrhea in five women with endometriosis conducted in our laboratory revealed that dysmenorrhea significantly improved without any adverse events, and the size of the endometriotic cysts significantly decreased (data not shown). These results prompted the need for large-scale validation studies to identify the efficacy and safety of AT-04 for pain relief in women with endometriosis.

## Methods

### Aim

The aim of this study is to explore the efficacy and safety of AT-04 in women with endometriosis-related pain.

### Design and setting

This is a phase III, sham-controlled, double-blind, parallel-group study. This study will be conducted in the Chiba University Hospital and its affiliated facilities, The University of Tokyo Hospital, University of Yamanashi Hospital, Fukuoka University, KASHIWAZAKI OB/GYN CLINIC, Iryohojinshadan Seijunkai Juno Vesta Clinic Hatta, TSUBAKI Women’s Clinic, and Yokosuka Haruko Lady’s Clinic. Details of the participating research facilities are provided in Additional file 1.

### Study participants

#### Inclusion criteria

Participants must fulfil the following inclusion criteria:

1. Premenopausal women aged ≥ 18 years at the time of obtaining consent
2. Patients clinically diagnosed with endometriosis meeting one of the following criteria (additional diagnosis required if the disease recurs after surgery):
  - laparoscopy or laparotomy performed within 5 years of the start of treatment
  - magnetic resonance imaging or ultrasonography (transvaginal, transabdominal, or transrectal) performed within 1 year prior to the start of treatment
  - pelvic and rectal examination performed prior to the start of treatment
3. Patients with dysmenorrhea or pelvic pain believed to be caused by endometriosis, with at least one assessed by the principal investigator to be moderate or higher on the Biberoglu and Behrman (B&B) rating scale before the start of treatment [10]
4. Patients with an average Numeric Rating Scale (NRS) score ≥ 4 for endometriosis-related pain within 28 days before obtaining consent [10]
5. Patients who have not initiated any new treatment for endometriosis or made changes to their existing treatment (including prescription details, dosage, and administration) within 28 days before obtaining consent
6. Patients who, based on the assessment of the principal investigator, show no evidence of acute endometriosis deterioration within 28 days before obtaining consent.
7. Patients who provide written consent to participate in the study.

#### Exclusion criteria

Patients meeting the following criteria will be excluded:

1. Use of the following drugs within 8 weeks prior to obtaining consent: clinical trial or investigational drugs, GnRH analogs, danazol and aromatase inhibitors, and selective estrogen receptor modulators
2. Previous use of alternating magnetic field therapy devices, including the study devices
3. Patients who routinely use non-steroidal anti-inflammatory drugs (NSAIDs)
4. Patients with ovarian endometriotic cysts > 10 cm in diameter and aged > 40 years
5. History of bilateral oophorectomy
6. Significant or unexplained irregular uterine bleeding determined by the principal investigator
7. Patients with uterine fibroids who may require new treatment during the study period in the opinion of the principal investigator
8. Irritable bowel syndrome and/or lower abdominal pain due to severe interstitial cysts
9. Patients with a history of, or complications of, severe hepatic disorder, jaundice, renal disorder, cardiovascular, endocrine system, metabolic, pulmonary, gastrointestinal, neurological, or urological diseases, immune disorders, psychiatric diseases (especially depression-like symptoms), and suicide attempts due to such disorders
10. Patients using life-supporting medical electrical equipment such as artificial heart lungs and pacemakers
11. Patients using medical electrical equipment, such as electrocardiographs
12. Patients participating in clinical trials or clinical studies on other drugs or medical devices
13. Patients requiring hospitalization for treatment

### Participant screening

Consent will be obtained from eligible participants 28–35 days before randomization. The examination items at screening will encompass the following items:

1. Participant characteristics at the baseline, including the date of consent, patient identification code, age, height (cm), weight (kg), body mass index (BMI), medical history, comorbidities, prior treatments, and physical findings, including major symptoms of endometriosis, gravidity, and parity
2. Vital signs, including systolic and diastolic blood pressure and body temperature
3. Evaluation of subjective and objective pain symptoms
4. Determination of the average NRS score for the 4 weeks preceding the visit
5. Evaluation of the B&B score
6. Ultrasound examination to identify ovarian endometriotic cysts and other endometriotic lesions. For assessment of ovarian endometriotic cysts, the maximum diameter of each cyst and their perpendicular diameter will be measured, and the calculated volume (cm^3^) will be recorded.
7. Monitoring the occurrence of adverse events
8. Documentation of concomitant medications and therapies.

### Discontinuation criteria

The study will be terminated if any of the following criteria are met during the study period:

1. Occurrence of unexpected severe illnesses or conditions, including physical disability or death, events that may lead to physical disability or death, hospitalization or extension of hospital stay, and congenital disorders or abnormalities in the next generation
2. Frequent occurrence of predictable severe conditions that significantly exceed expectations
3. Serious adverse events for which a causal relationship cannot be excluded
4. Reports indicating significant changes in the incidence, frequency, and conditions of disease occurrence
5. Reports indicating the potential occurrence of cancer, other serious diseases, disabilities, or death
6. Information suggesting a lack of efficacy in the trial
7. Information on measures implemented to prevent manufacturing, importation, or sale, as well as recall, disposal, or other actions to prevent the occurrence or spread of health and hygiene hazards for commercial products having the same effect as the tested devices.

### Study device

**Fig. 1:**
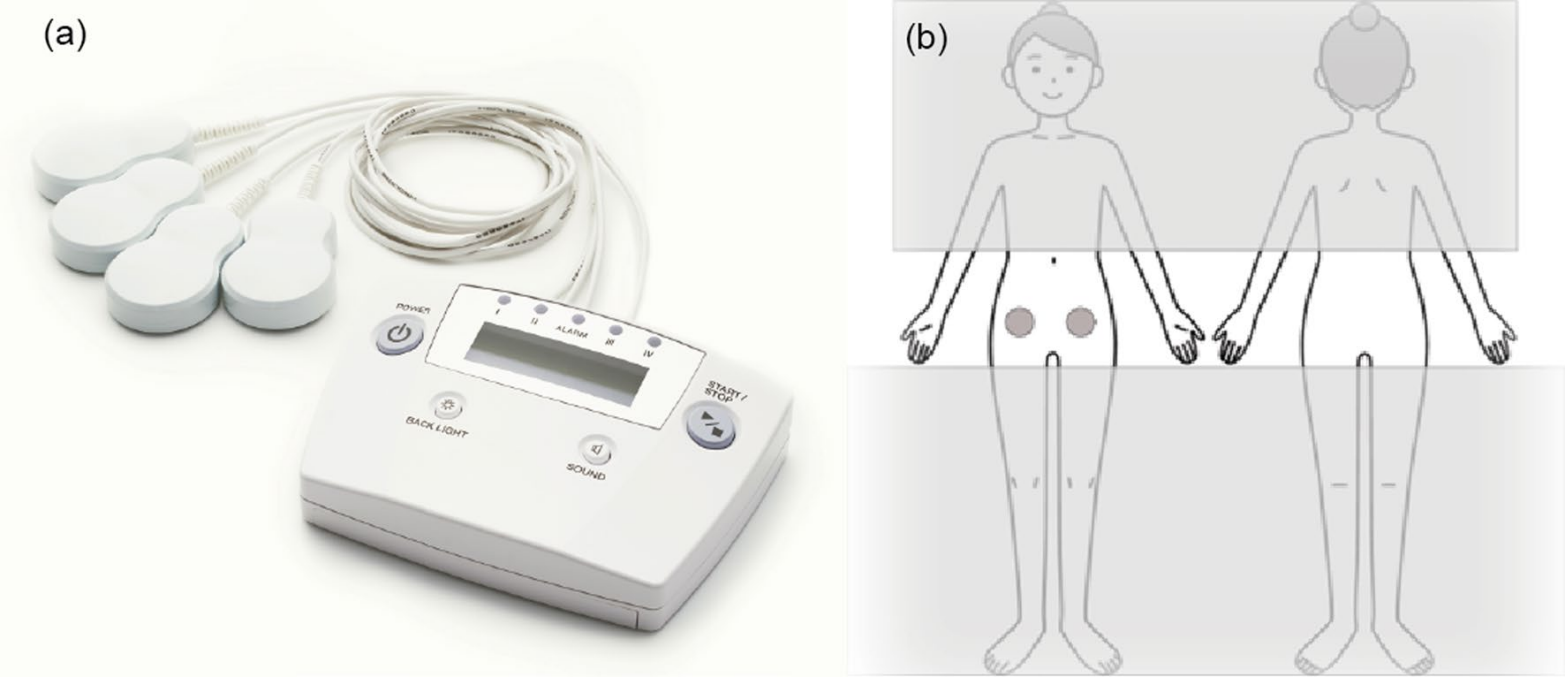
Study device and location for applying pads. **a** Actual photo of the AT-04. The appearance of the test device (AT-04) and the sham device (S-02) is identical, and it is impossible to distinguish the two based on their external features. **b** Location for applying pads. Participants will attach the pads themselves to both sides of their lower abdomens. The grey circle indicates the basic location for applying the pads

The AT-04 is a minimally invasive device comprising a controller and a dual-coil emitter assembly powered by a 3.7-V battery (Fig. 1a). The dual emitter simultaneously generates alternating magnetic fields. Detailed instructions, in Japanese, for using the device are provided in the Additional file. Briefly, participants are instructed to attach two pads to the left and right sides of the lower abdomen and then press the start button to generate local electromagnetic waves (Fig. 1b). The device automatically stops after 30 min. If participants experience pain in areas other than those mentioned above, they can attach the remaining two pads to the affected area(s). The test and sham devices will be provided free of charge by Peace Mind Co. Ltd., Kumamoto, Japan.

### Interventions

**Fig. 2:**
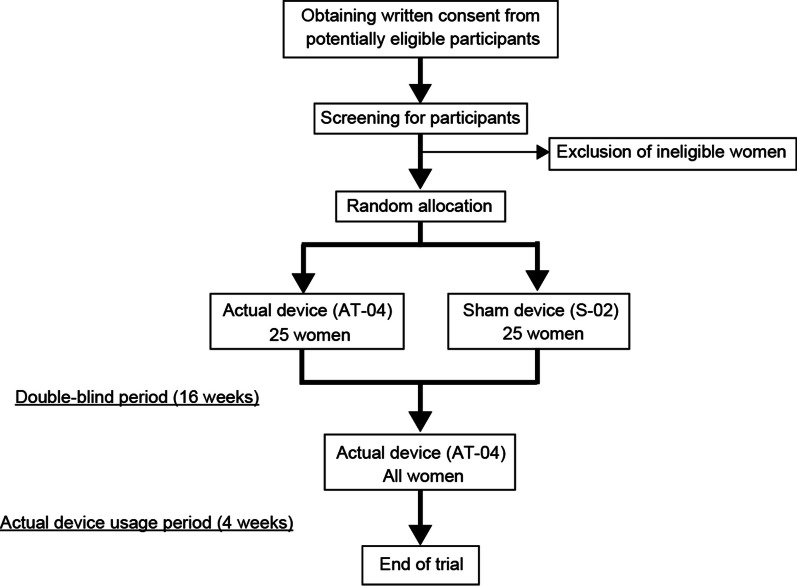
Overview of the study design. After providing consent, eligible women will be randomly assigned to the treatment group, utilizing AT-04, or the control group, utilizing S-02. Participants will use these devices for 16 weeks (double-blind period), after which they will use AT-04 for an additional 4 weeks (actual device usage period)

The study design is shown in Fig. 2. After obtaining consent, eligibility for this study will be confirmed through screening tests. Central enrolment and randomized assignment will be conducted at the data center using a 1:1 ratio to minimize bias. The actual treatment group will use the AT-04 and the control group will use the S-02, a sham device, for 16 weeks. At the 16-week visit, participants will be asked to bring their device for replacement with a new device (all actual devices). To ensure blinding after replacement with the actual device at week 16, participants will be informed, at the time of obtaining their consent to participate, that the feeling of use may vary depending on the device under study. The pads will be applied to at least two sites on the lower abdomen, including the uterine and ovarian areas; if there are other painful areas, two additional pads (a maximum of four sites) will be applied to those areas.

### Outcome measures

#### Primary outcome

The primary outcome of this study is the change in the NRS score for endometriosis-related pain collected at each visit and at the end of the double-blind period (after 16 weeks) compared with the baseline score at the start of treatment.

#### Secondary outcomes

The secondary outcomes of this study are summarized in Table 1. Measurements will be conducted at the baseline and at 4, 6, 12, 16, and 20 weeks after treatment initiation. Longitudinal pain during the study period will be recorded using electronic patient-reported outcomes (ePRO). The B&B scale, a specialized scoring system for evaluating endometriosis-related pain, will be employed. It consists of a rating based on the patient’s self-assessment of pelvic pain, dysmenorrhea, and dyspareunia (Bourdel et al. 2014). The quality of life of the participants will be evaluated using the Endometriosis Health Profile-30 (EHP-30) and the EuroQol 5-Dimension (EQ-5D) Health-Related Quality of Life (HRQoL) Questionnaire.

**Table 1:** Secondary outcomes in this study

Outcomes		Evaluation tools	Time points
Efficacy for pain	Pelvic pain	NRS score	Comparison baseline* with 4, 8, 12, and 20 weeks after treatment initiation
	Pain during menstruation#	NRS score by ePRO^$^	From treatment initiation to end of treatment
	Pain other than menstruation	NRS score by ePRO^$^	From treatment initiation to end of treatment
	Chronic pelvic pain not related to menstruation, dysmenorrhea, and dyspareunia	Biberoglu & Behrman (B&B) scale	Comparison baseline* with 4,8,12, and 20 weeks after treatment initiation
Quality of life		Endometriosis Health Profile-30 (EHP-30) score	Comparison baseline* with 4, 8, 12, and 20 weeks after treatment initiation
		Health-related quality of life (EQ-5D)	Comparison baseline* with 4, 8, 12, and 20 weeks after treatment initiation
Efficacy for endometriosis lesions	Size of ovarian endometriotic cysts	Transvaginal ultrasound	Comparison baseline* with 16 weeks after treatment initiation**
Safety of the device	The frequency and proportion of malfunctions and adverse events in the test device		

*Baseline was defined as a point before treatment initiation

^#^Pain during menstruation including menstrual pain and other pelvic pain

^$^eRPO: Electronic Patient-Reported Outcomes; NRS: Numeric Rating Scale

**Time at the end of the treatment period

#### Observation, examination, and evaluation items at each visit (Table 2)

**Table 2:**
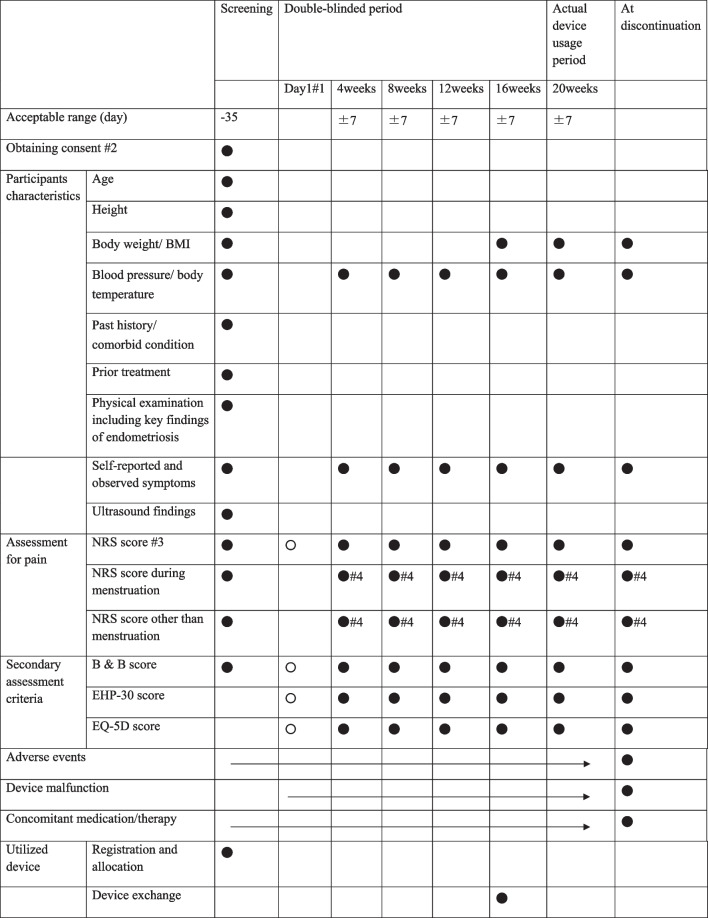

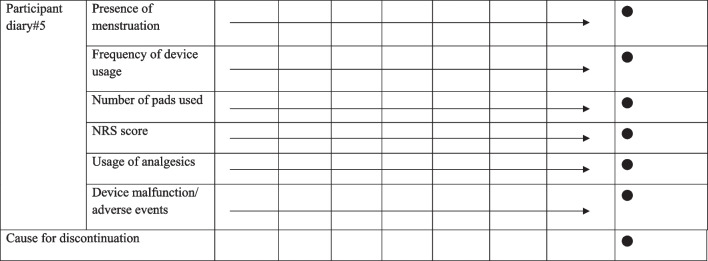
Study schedule for observation, inspection, and evaluation

● Represents the examination and assessment items to be performed at each visit

^#^1: Acceptable range of the required tests at Day 1, as indicated by 〇, are 14 days. Treatment using test device starts at Day 1

^#^2: Consent from the participants must be obtained at least 28 days before the start of the study

^#^3: Participants recall and assess the level of pain experienced over the past 4 weeks at each visit

^#^4: Data collected through the electronic Patient Reported Outcome (ePRO) system will be used for the assessment

^#^5: The participant diary will start recording from the date of consent. Recordings until the day before Day1 will be on paper diary, while from Day1 onwards, record will be collected by ePRO

BMI: body mass index; NRS: Numeric rating Scale; B&B: Biberoglu and Behrman; EHP-30: Endometriosis Health Profile-30; EQ-5D: EuroQol 5-Dimension

Treatment using the test device will commence on Day 1, and the NRS, B&B, EHP-30, and EQ-5D scores will also be collected on Day 1. NRS scores will be assessed by participants recalling their endometriosis-related pelvic pain over the 4 weeks prior to the visit. The following items will be assessed at the visits in weeks 4, 8, 12, 16, and 20 after the initiation of the trial:

1. Height and BMI
2. Vital signs, including systolic and diastolic blood pressure and body temperature
3. Subjective and objective pain symptoms
4. Average NRS score for the 4 weeks preceding the visit
5. Maximum and average NRS scores during menstruation within the 4 weeks preceding the visit
6. Maximum and average NRS scores for periods other than menstruation within the 4 weeks preceding the visit
7. B&B, EHP-30 score, and EQ-5D scores
8. Ultrasonography to identify ovarian endometriotic cysts and other endometriotic lesions. Ovarian endometriotic cysts will be evaluated using the same measurements as the screening examination
9. Occurrence of adverse events
10. Concomitant medications and therapies.

In the event of discontinuation of the device by a participant, the reasons for discontinuation will be included in addition to the observation items mentioned above.

#### Participant diary

Paper-based records and ePRO will be used to collect daily data, including the presence or absence of menstruation, number of devices used, quantity of device pads used, NRS scores (reflecting maximum pain in the preceding 24 h before input), utilization of analgesics, and the occurrence of adverse events.

### Assignment of interventions and blinding

After obtaining consent, eligible participants confirmed through screening tests will be randomized. The randomization at a 1:1 ratio will be conducted at the data center to achieve central registration and minimize bias. For the random allocation, the minimization method will be employed, adjusting for the trial facility and the presence or absence of low dose estrogen-progestin and progestin treatment within each group. The principal investigator will initiate protocol treatments for participants determined to be ‘eligible’ according to the randomization results. Therefore, the trial participants and principal investigator will be blinded to interventions after assignment.

### Statistical analysis

The baseline characteristics for participants in each group will be summarized, presenting frequencies and percentages for categorical variables and summary statistics (number of cases, mean, standard deviation, and minimum, median, and maximum values) for continuous variables. For group comparisons, Pearson’s Chi-squared test will be used for categorical variables, Fisher’s exact test will be applied for cells with an expected frequency < 5% and > 20%, and the t-test or Mann–Whitney U test will be used for continuous variables.

A two-tailed 5% significance level will be used. A two-tailed Student’s t-test will assess the primary outcome (null hypothesis: the difference in NRS score change between the treatment and control groups equals zero; alternative hypothesis: the difference in NRS score change between the treatment and control groups is not equal to zero). Rejection of the null hypothesis and adoption of the alternative hypothesis will occur if p < 0.05.

Additionally, an analysis of variance with the allocation factor as a fixed effect will be performed as a sensitivity analysis, and the Wilcoxon rank-sum test will be performed for a non-parametric analysis. The secondary endpoint analysis will be similar to the primary endpoint analysis. For the safety endpoint, frequencies and proportions of the patients with adverse events will be presented for each group. Exact two-sided 95% confidence intervals, assuming a binomial distribution, will be calculated for each group, and Fisher’s exact test will be used to compare groups. All statistical analyses will be performed using SAS version 9.4 or higher (SAS Institute Japan Ltd, Tokyo).

### Sample size calculation

The sample size calculation is based on the findings of a randomized controlled trial assessing the effect of the AT-02, a prototype of the AT-04, in patients with fibromyalgia [9]. Anticipated placebo effects of the sham device are approximately 14%. In previous open trials for dysmenorrhea, participants with NRS scores ≥ 6 before treatment initiation experienced a significant reduction in NRS scores by 1.73 as the total effect, encompassing the true effect and placebo effect. Assuming a true effect of 1.49 and attributing 14% as the placebo effect, the calculated placebo effect will be 0.24. The standard deviation of the actual device was approximately 1.8, and that of the sham device was approximately 0.3. Employing a two-sided non-paired t-test, the effect size was set to 0.969, alpha was set to 0.05, and power was set to 0.9. A sample size of 24 participants per group, totalling 48 participants, is required to achieve a conservative beta error. Considering potential dropouts, a sample size of 50 is deemed appropriate.

### Data management

The principal investigator and collaborating physicians will utilize Electronic Data Capture (EDC) to produce, manage, and revise the study report. External data collection will be facilitated through ePRO. The source materials include: (1) informed consent forms and documents providing information to the study participants; (2) documents containing participant baseline data, including patient charts, nursing documents, laboratory results, and imaging results; (3) documents on the use of testing devices; and (4) documents and records related to necessary tests under the Clinical Trials Act of Japan.

### Monitoring

Considering the study’s risk profile, both on-site and off-site monitoring will be carried out in adherence to the quality control protocols of the facilities. Monitoring personnel are required to compile reports on significant findings, including diseases and noncompliance, or provide summaries of the factual circumstances. Importantly, they must refrain from disclosing information acquired during their duties without valid reasons.

## Discussion

This randomized trial aims to evaluate the efficacy of AT-04, a novel pain management device, in treating endometriosis-related pain. The AT-04 generates weak alternating magnetic fields, providing effective local pain relief. Women who have undergone standard hormonal treatment for endometriosis at baseline will be enrolled. This allows for assessment of whether the device remains effective when used in conjunction with existing treatment methods. Additionally, recruitment of women who have not undergone existing treatments can be challenging.

Current therapies for endometriosis-related pain have several advantages and disadvantages. NSAIDs are the first-choice analgesics, and traditional Chinese medicines are frequently used for pain control [11]. While these drugs have few side effects and are generally well tolerated, their analgesic effects vary among individuals. Hormonal treatments using low-dose OC, progestins, and GnRH analogs have demonstrated efficacy in improving endometriosis-related pain [12]. However, continuous use of hormonal treatments may be challenging if women wish to become pregnant due to impaired ovulation. Moreover, each hormonal treatment has specific adverse effects. The use of OCs is associated with pulmonary embolism and deep venous thrombosis. Long-term use of GnRH analogs is restricted due to the significant loss of bone mineral density. Dienogest administration may lead to persistent abnormal uterine bleeding, impacting a woman’s quality of life. While laparoscopic surgeries are frequently performed to remove endometriotic lesions, a significant number of recurrences have been observed. The loss of the ovarian reserve after laparoscopic excision of ovarian endometriotic cysts negatively affects women with infertility [13].

Approximately one-third of women with endometriosis-related pain experience poor pain control, which affects their daily lives. Consequently, several therapeutic options have been developed to alleviate pain through diverse mechanisms. Oral GnRH antagonists such as relugolix, elagolix, and linzagolix have proven effective for short-term endometriosis-related pain [14]. Combination therapy with relugolix and low-dose estrogen and progestin has shown significant improvement in endometriosis-related pain without serious adverse events [15]. Supplementation of antioxidant vitamins has been effective in significantly reducing inflammatory markers and pelvic pain scores in women with endometriosis [16]. Additionally, a combination of N-acetyl cysteine, alpha-lipoic acid, bromelain, and zinc, which have antioxidant action upstream of the cyclooxygenase-2 pathway, has shown effectiveness in controlling endometriosis-related pain [17]. Although opioids are generally not recommended for pain relief in women with endometriosis, opioid prescriptions have been identified for women diagnosed with endometriosis within the past year in the United States [18]. Women with endometriosis have a four-fold greater risk of chronic opioid use compared to those without endometriosis [19]. Psychological interventions, such as cognitive-behavioral therapy, mindfulness therapy, yoga, psychoeducation, and progressive muscular relaxation, significantly reduce endometriosis-related pain [20]. Chinese acupuncture has also demonstrated effectiveness in pain control for endometriosis [21].

Improving the HRQoL of women with endometriosis is another important aspect of patient-centered narrative medicine. Therefore, this study aims to evaluate HRQoL using the EHP-30 and EQ-5D scoring systems. The EHP-30 is a valid and reproducible measure of HRQoL in women with endometriosis, consisting of 30 questions divided into five categories: pain, control and powerlessness, social support, emotional well-being, and self-image [22, 23]. The EQ-5D is a generic instrument for describing HRQoL in daily life, evaluating daily life in five dimensions: mobility, self-care, usual activities, pain/discomfort, and anxiety/depression [24].

This study has some potential limitations that need consideration. First, it is challenging to determine the sole efficacy of AT-04 in treating endometriosis-related pain since all eligible women would have already been diagnosed with endometriosis, and most would have undergone treatment for endometriosis. Second, the NRS and HRQoL measurements may vary during the study period due to hormonal fluctuations associated with the menstrual cycle; this may impact the assessment of pain and quality of life. Finally, there is a possibility of insufficient pain relief by AT-04 because the study targets women with an NRS score ≥ 4 with moderate endometriosis-related pain. The effectiveness of pain relief may be comparatively weak in women with moderate pain. Furthermore, this study will be held in Japan. The relevance to other countries, race, ethnicities and cultures is another limitation.

## Conclusion

In summary, this randomized study has the potential to develop a novel method of managing endometriosis-related pain. The AT-04 is an ultralow-invasive device that can be used without inhibiting ovulation in women with endometriosis. The mechanism of pain management using the AT-04 is distinctly different to that of existing treatments; this suggests potential efficacy in the treatment of women with endometriosis-related pain.

### Supplementary Information

**Additional file 1.** The study protocol has been approved by the Clinical Study Review Board of Chiba University Hospital, Chiba, Japan, and registered by the Japan Registry of Clinical Trials (jRCTs032230278, https://jrct.niph.go.jp/). An overview of this research is publicly available through the Japan Registry of Clinical Trials.

**Additional file 2.** The participant consent form has been approved by the Clinical Study Review Board of Chiba University Hospital, Chiba, Japan, and registered by the Japan Registry of Clinical Trials (jRCTs032230278, https://jrct.niph.go.jp/). An overview of this research is publicly available through the Japan Registry of Clinical Trials.

## Data Availability

The detailed study protocols and a participant consent form, which are described in Japanese, are available in the Additional file 1 and 2, respectively.

## Abbreviations

AT-04: Angel Touch device 04
BMI: Body mass index
B&B: Biberoglu and Behrman
EDC: Electronic data capture
EHP-30: Endometriosis Health Profile-30
ePRO: Electronic patient-reported outcomes
EQ-5D: The EuroQol 5-dimension
GnRH: Gonadotropin-releasing hormone
HRQoL: Health-related quality of life
NRS: Numeric Rating Scale
NSAIDs: Non-steroidal anti-inflammatory drugs
OC: Oral contraceptive
