# Supplementary material for: Efficacy and safety of a novel pain management device, AT-04, for endometriosis-related pain: study protocol for a phase III randomized controlled trial
Source: Reprod Health. 2024 Jan 26;21:12. doi: 10.1186/s12978-024-01739-8 (PMC10811886; doi:10.1186/s12978-024-01739-8)
Supplement: Supplementary file 1 — Additional file 1. The study protocol has been approved by the Clinical Study Review Board of Chiba University Hospital, Chiba, Japan, and registered by the Japan Registry of Clinical Trials (jRCTs032230278, https://jrct.niph.go.jp/). An overview of this research is publicly available through the Japan Registry of Clinical Trials. [file 12978_2024_1739_MOESM1_ESM.pdf]

研究計画書

子宮内膜症に対する **AT-04** の有効性及び安全性に関する

シャム機対照二重盲検並行群間比較試験

第III相試験

版数：5.0 版
試験管理番号 CRB0070-23
作成日：2023 年 10 月 25 日

改訂履歴

作成日	版数
2022 年 12 月 25 日	0.1 版（初版）
2023 年 2 月 6 日	0.2
2023 年 3 月 31 日	0.3
2023 年 4 月 14 日	1.0（CRB 申請版）
2023 年 6 月 13 日	2.0
2023 年 7 月 7 日	3.0
2023 年 8 月 25 日	3.1
2023 年 10 月 6 日	4.0
2023 月 10 月 25 日	5.0

本研究計画書における略語および用語の定義

5-HT	5-ヒドロキシトリプタミン（5-hydroxytryptamine）
ACHES	A：Abdominal pain；腹痛，C：Chest pain；胸痛・突然の息切れ，H：Headache；激しい頭痛，E：Eye/speech problem；急性視力障害・構語障害，S：Severe leg pain；下肢の疼痛・浮腫）
B&B	Biberoglu & Behrman
COI	Conflict of interest（利益相反）
CRF	Case report form（症例報告書）
CTCAE	Common terminology criteria for adverse events（有害事象共通用語規準）
DPC	Diagnosis Procedure Combination（入院費の包括払い方式）
EDC	Electric Data Capture（試験データの電子化システム）
EHP-30	Endometriosis Health Profile-30
FAS	full analysis set
GCP	Good clinical practice（医薬品の臨床試験の実施に関する基準）
GnRH	GnRH（gonadotropin releasing hormone，ゴナドトロピン放出ホルモン）
jRCT	Japan Registry of Clinical Trials
LEP	Low dose Estrogen Progestin Combination（低用量エストロゲン・プロゲスチン配合剤）
LNG-IUS	Levonorgestrel Intra Uterine System（黄体ホルモンであるレボノルゲストレル（LNG）を子宮の中に持続的に放出する子宮内システム）
MAO	モノアミン酸化酵素（Monoamine oxidase）
NGF	Nerve growth factor（神経成長因子）
NRS	Numeric Rating Scale（疼痛スケール）
NSAIDs	Non-Steroidal Anti-Inflammatory Drug（非ステロイド性抗炎症薬）
PMDA	Phamaceuticals and Medical Devices Agency（独立行政法人医薬品医療機器総合機構）
PPS	per protocol set
PS	Performance Status（パフォーマンスステータス、全身状態）
QOL	Quality of Life（生活の質）
RCT	Randomized controlled trial（ランダム化比較試験）
SP	Safety population（安全性解析対象集団）
TENS	Transcutaneous electrical nerve stimula
VTE	venous thromboembolism

＜目次＞

0. 試験の概要 6
1. 研究の背景 9
1.1. 国内外における対象疾患の状況 9
1.2. これまでに実施されてきた標準治療の経緯及び内容 10
1.3. 現在の標準治療の内容及び治療成績（臨床試験成績を含む） 10
1.4. 当該臨床研究の必要性につながる、現在の標準治療の課題、不明点等 12
1.5. 当該臨床研究に用いる医薬品等に関する情報 13
2. 試験の目的 13
3. 対象疾患 13
3.1. 選択基準 13
3.2. 除外基準 14
4. 被験者の同意 15
4.1. 同意文書及びその他の説明文書の作成並びに改訂 15
4.2. 同意取得の時期と方法 15
4.3. 被験者に対する説明事項 15
5. 試験のデザイン 17
5.1. 評価項目 17
5.2. 試験デザインの概略 17
5.3. 試験の方法 17
5.4. 目標被験者数と試験実施期間 18
5.5. 症例登録・割付方法 18
5.5.1. 施設登録 18
5.5.2. 症例登録・割付方法 18
5.5.3. 割付調整因子 18
5.5.4. 症例登録先 18
5.6. 不適格となった被験者の取り扱い 19
5.7. 試験機器の施術スケジュールおよび機器の使用方法 19
5.8. 休止（中断）及び再開の基準 19
5.9. 機器使用中止基準 19
5.9.1. 被験者の妊娠 20
5.10. 個々の症例の中止基準 20
5.11. 併用薬及び併用療法 20
5.11.1. 併用制限薬 20
5.11.2. 併用禁止薬 20
5.12. 併用禁止療法 21
5.13. 試験終了後の対応 21
6. 試験機器 22
6.1. 試験機器の概要 22
6.2. 試験機器の管理方法 22
7. 観察・検査・評価項目、方法及び実施時期 23
7.1. 実施スケジュールと手順 23
7.1.1. スクリーニング 24
7.1.2. 観察・検査・評価項目 24
7.1.2.1. Day1（0 週） 24

7.1.2.2. 4週後 24
7.1.2.3. 8週後 25
7.1.2.4. 12週後 25
7.1.2.5. 16週後 25
7.1.2.6. 20週後 26
7.1.2.7. 中止時 26
7.2. 患者日誌 26
7.3. 評価の方法 27
7.3.1. NRS スコア 27
7.3.2. B&B スケール 27
7.3.3. EHP-30 スコア 28
7.3.4. EQ-5D 28
8. 安全性の評価に関する取扱い 30
8.1. 有害事象及び不具合の定義 30
8.2. 疾病等の定義 30
8.3. 重篤な有害事象 30
8.4. 非重篤な有害事象 30
8.5. 報告の対象となる有害事象及び不具合 30
8.6. 有害事象の回復性と試験機器との因果関係 30
8.7. 有害事象の評価に必要な情報 30
8.8. 不具合の評価に必要な情報 31
9. 疾病等及び重篤な有害事象発生時 31
9.1. 有害事象発生時の対応 31
9.2. 疾病等及び重篤な有害事象発生時の対応 31
9.3. 疾病等及び重篤な有害事象報告の手順 31
10. 統計学的事項 34
10.1. 解析対象集団 34
10.1.1. 最大の解析対象集団 (full analysis set：FAS) 34
10.1.2. 研究計画書に適合した対象集団 (per protocol set：PPS) 34
10.1.3. 安全性解析対象集団 34
10.2. 目標症例数と設定根拠 34
10.3. 症例の取り扱い 35
10.4. データの取り扱い 35
10.5. 統計解析項目および解析計画 35
10.5.1. 被験者背景の解析 35
10.5.2. 有効性の解析 35
10.5.2.1. 主たる解析 35
10.5.2.2. 副次解析 35
10.5.3. 安全性の解析 35
10.5.4. 探索的解析 36
10.5.5. 中間解析 36
10.6. 独立データモニタリング委員会 36
10.7. 最終解析 36
11. 研究計画書の遵守および不適合 36
12. 研究計画書、同意説明文書又は解析計画に関する変更 36

12.1. 研究計画書および同意説明文書の改訂 36
12.2. 統計解析計画の変更 37
13. 試験の中止または終了 37
13.1. 試験全体での中止の基準 37
13.2. 試験全体での中止する場合の手続き 37
13.3. 試験の終了 37
14. データマネジメント 38
14.1. データ登録の方法及び管理方法 38
14.2. 症例報告書に直接記載され、かつ原資料(原データ)と解すべき資料の特定 38
15. 原資料及びその他の記録の保存 38
16. 試料・情報等の保存及び他機関等の試料等の利用 39
17. 原資料の直接閲覧 39
18. 試験の品質管理及び品質保証 39
18.1. 品質管理 39
18.2. 品質保証 39
19. 倫理 40
19.1. 本研究の参加に伴って予想されうる利益 40
19.2. 本研究の参加に伴って予想されうる不利益 40
19.3. 総合的な評価 40
20. 被験者の秘密の保全 40
21. 認定臨床研究審査委員会の審査等 40
22. 健康被害補償及び保険 41
22.1. 健康被害の補償 41
22.2. 賠償保険への加入 41
22.3. 臨床研究保険（補償保険）への加入 41
23. 金銭の支払い 41
24. 研究資金および利益の衝突 41
25. 研究に関する情報公開 42
26. 結果の公表 42
26.1. 公表の方法 42
26.2. 公表についての取り決め 42
27. 試験実施体制 43
28. 参考資料・文献リスト 46

0. 試験の概要

**試験課題名**	子宮内膜症に対する AT-04 の有効性及び安全性に関するシャム機対照二重盲検並行群間比較試験
**試験の目的**	子宮内膜症患者において、AT-04 照射群とシャム機（非照射）群との NRS 値の投与前後における変化量を群間で比較し、AT-04 照射による有効性を検討する。また、不具合、有害事象の発現率を群間で比較し、AT-04 照射の安全性についても検討する。
**試験デザイン**	検証的、並行群間、ランダム化、二重盲検、実機対照試験、多施設共同
**フェーズ**	第III相試験
**被験機器**	交番磁界治療器<br>（１）類別名称：理学診療用器具<br>（２）クラス分類：管理医療機器・特定保守管理医療機器<br>（３）販売名：エイト<br>（４）承認・認証・届出番号：承認番号：30400BZX00015000<br>（５）使用目的又は効果： 本品は、2 種類の交番磁界を経皮的に照射し、神経を刺激することで疼痛を緩和させるために用いられる。<br>（６）使用方法： 添付文書又は取扱説明書参照<br>（７）製造販売業者等：株式会社Ｐ・マインド<br>（８）その他：添付文書参照又は取扱説明書参照
**対象疾患**	子宮内膜症
**選択基準**	以下の選択基準を全て満たす者を対象とする。<br>（１）同意取得時に、18 歳以上の患者<br>（２）閉経前の女性患者<br>（３）子宮内膜症患者（以下のいずれかに該当する者とする。なお、術後再発した場合は、再度、以下のいずれかで診断されていること）<br>①治療開始前の 5 年以内に実施した開腹又は腹腔鏡検査により子宮内膜症と診断された患者<br>②治療開始前の 1 年以内に実施した MRI または治療開始前に実施した超音波検査（経腟、経腹又は経直腸）により卵巣チョコレート嚢胞等画像上検出される子宮内膜症を認める患者<br>③治療開始前に実施した内診・直腸診によりダグラス窩の硬結、子宮可動性の制限または骨盤の圧痛のいずれかを認め、臨床子宮内膜症と診断される患者<br>（４）子宮内膜症に由来すると考えられる月経困難症または骨盤痛を有し、治療開始前にて B&B 評価スケールで少なくとも一方が中等度以上であると研究責任（分担）医師が判断した患者<br>（５）同意取得前の 28 日間において、子宮内膜症による痛み NRS（Numeric Rating Scale）スコアの平均が 4 以上である患者<br>（６）同意取得前の 28 日間において、子宮内膜症に対する治療の新たな開始、もしくは治療内容（薬の処方内容・用法・用量を含む）の変更がない患者<br>（７）同意取得前の 28 日間において、子宮内膜症の急性憎悪を疑う所見がないと研究責任（分担）医師が判断した患者<br>（８）本人の自由意思により、研究の参加について文書による同意を得られている患者

**除外基準**	以下の除外基準に 一つでも該当する者は対象から除外する。<br>（１）以下の医薬品等を同意取得の 8 週前以内に使用した患者<br>・臨床試験薬または治験薬<br>・GnRH アナログ製剤、ダナゾール及びアロマターゼ阻害剤、選択的エストロゲン受容体モジュレーター製剤<br>（２）試験機器を含む交番磁界治療機器を過去に使用したことのある患者<br>（３）NSAIDs を定期的に服用している患者<br>（４）卵巣チョコレート嚢胞が 10 cm 以上、かつ年齢が 40 歳以上の患者<br>（５）両側卵巣摘出術の既往がある患者<br>（６）著しい不正子宮出血、原因不明の不正子宮出血の合併があると研究責任（分担）医師が判断した患者<br>（７）試験期間中に新たに治療を要すると研究責任（分担）医師が判断する子宮筋腫を合併している患者<br>（８）過敏性腸症候群および・または重度の間質性膀胱炎による下腹部痛を有する患者<br>（９）重度の肝障害、黄疸、腎障害、心血管系疾患、内分泌系疾患、代謝疾患、肺疾患、胃腸疾患、神経疾患、泌尿器疾患、免疫疾患、並びに精神疾患（特にうつ様症状）およびそれに起因する自殺企図等の既往または合併がある患者<br>（１０）人工心肺及びペースメーカ等の生命維持用医用電気機器を使用している患者<br>（１１）心電計等の装着型医用電気機器を使用している患者<br>（１２）他の医薬品や医療機器の治験や臨床試験に参加している患者<br>（１３）治療のために入院の必要がある患者
**評価項目**	**主要評価項目**<br>治療開始前のベースラインと比較した、二重盲検期間終了時（16 週後）における子宮内膜症関連疼痛の各来院時に収集した思い出しによる Numeric Rating Scale (NRS)スコアの変化量<br><br>**副次評価項目**<br>【有効性の副次評価項目】<br>● 治療開始前のベースラインと比較した、4,8,12,20 週後における子宮内膜症関連疼痛の各来院時に収集した思い出しによる Numeric Rating Scale (NRS)スコアの変化量<br>● 治療開始前のベースラインと比較した、治療期間中における月経痛および月経期間の子宮内膜症骨盤痛の ePRO において収集した毎日の NRS スコアの変化量<br>● 治療開始前のベースラインと比較した、治療期間中における月経期間以外の子宮内膜症骨盤痛の ePRO において収集した毎日の NRS スコアの変化量<br>● 治療開始前のベースラインと比較した、4,8,12,16,20 週後の Biberoglu & Behrman (B&B) スコアの変化量<br>● 治療開始前のベースラインと比較した、4,8,12,16,20 週後の Endometriosis Health Profile-30 (EHP-30) スコアの変化量<br>● 治療開始前のベースラインと比較した、4,8,12,16,20 週後の健康状態包括的尺度 (EQ-5D)の変化量<br>● 治療開始前のベースラインと比較した、二重盲検期間終了後（16 週後）の卵巣チョコレート嚢胞の体積の変化量<br><br>【安全性の副次評価項目】

	試験機器の不具合及び有害事象の発現頻度・割合
**試験方法**	本試験は、二重盲検期（16 週間）、実機使用期（4 週間）の 2 期間で構成される。同意取得後、スクリーニング検査にて適格性を確認し、データセンターにおいて中央登録及びバイアスを最小化するための無作為化割付を 1：1 で行う。（詳細は「5.5 症例登録・割付方法」に記載）<br>実機照射群には、AT-04 を 16 週間施術する。また、対照群にはシャム機である S-02 を 16 週間施術する。その後、全症例に対し新規に実機である AT-04 を 4 週間施術する。
**目標被験者数**	50 名 (各群 25 名)
**試験実施期間**	試験実施期間： jRCT 公表日 - 2025 年 3 月 31 日<br>症例登録期間： jRCT 公表日 - 2024 年 3 月 31 日
**試験施設数**	8 施設
**倫理指針**	本試験の実施に際しては「ヘルシンキ宣言」に基づく倫理的原則、「臨床研究法」 及びその他の関連する規制要件を遵守するものとする。
**認定臨床研究審査委員会**	認定臨床研究審査委員会は、本試験が対象者の生命、健康及び人権を尊重するものであり、下記の基本理念に基づいて実施されることを審議する。<br>1) 社会的及び学術的意義を有する臨床研究を実施すること<br>2) 臨床研究の分野の特性に応じた科学的合理性を確保すること<br>3) 臨床研究により得られる利益及び臨床研究の対象者への負担その他の不利益を比較考量すること<br>4) 独立した公正な立場における審査意見業務を行う認定臨床研究審査委員会の審査を受けていること<br>5) 臨床研究の対象者への事前の十分な説明を行うとともに、自由な意思に基づく同意を得ること<br>6) 社会的に特別な配慮を必要とする者について、必要かつ適切な措置を講ずること<br>7) 臨床研究に利用する個人情報を適正に管理すること臨床研究の質及び透明性を確保すること

1. 研究の背景

1.1. 国内外における対象疾患の状況 [1]

子宮内膜症は生殖年齢女性の約10%に認められる慢性の進行性炎症疾患である。10代後半から20代前半に発症し、20代後半から30代前半に症状があらわれるのが一般的とされ、月経困難症・慢性痛などの疼痛によるQOLの低下が大きな問題となっている。

さらには、症状の進行により、不妊の原因（排卵障害・卵管障害)、妊娠できた際にも合併症（早産・妊娠高血圧腎症）が増加することに加え、卵巣がんへの進展を起こすことも知られ、少子化対策・女性活躍推進が叫ばれる昨今の社会情勢において重要な課題となっている疾患の1つである。子宮内膜症の標準治療については、大きく分けて薬物療法と手術療法がある。薬物療法は、第一選択としては鎮痛剤・漢方薬が使用されるが無効なことが多く、低用量ピル（低用量エストロゲン・プロゲスチン配合薬)、GnRHアナログ、黄体ホルモンなどのホルモン療法が選択される場合が多い。

手術療法は、妊娠の希望がある場合には、病巣部のみを切除し、子宮や卵巣の正常部分を残す保存手術が選択され、妊娠の希望がない場合には、病巣とともに子宮・卵巣および卵管などを摘出する根治手術が選択される。ただし、これらの現行の薬物療法は効果が不十分のことが多く、またホルモン治療は副作用出現率が高く、さらに治療中は妊娠が不可能であることから、長期管理が困難な場合が多い。

手術療法においても、保存手術を選択した場合は再発率が高く、根治手術を施行すると妊孕能が犠牲になるという問題点があり、やはり治療に難渋する場面が多い。

特に疼痛については、薬物療法・手術療法を持ってしても治療抵抗性であることが多い。これは本症の病理学的背景として、病巣周囲に神経増殖因子などの発現が亢進しており、知覚神経が増殖していること、疼痛が持続することで下行性疼痛抑制経路が低下、もしくは疼痛閾値が下がることが関連していると考えられており、病巣の制御よってもこれらがかならずしも改善しないことに起因すると考えられている。さらにこれらの疼痛が増悪することで時間的空間的に疼痛が拡大し、不安・抑うつ等の精神症状を誘発し、ひいては患者の社会生活が困難になる等QOLが著しく低下することにつながっていく。

本研究で用いる試験機器（AT-04）は、人体にほぼ無害な2kHz及び83.3MHzの微弱な交番磁界（磁界エネルギーは地磁気の約1/3程度）を同時発生させる超低侵襲的治療機器であり、患部にあてることにより痛みの緩和・治療効果を実現するものである。

本機器が疼痛改善効果を発揮する機序としては、過去のマウス等を用いた非臨床試験の結果より、神経成長因子の調整、末梢における炎症性サイトカインの抑制および下行抑制系の賦活化であると考えられており、上述のような本症の病理学的背景に鑑みると、本機器が子宮内膜症の病巣やそれに起因する疼痛に有効な可能性は十分期待できる[2]。実際、本機器の臨床試験では、すでに線維筋痛症[3]、腰痛症などで有効性かつ有害事象が発生しないという安全性を示しているだけでなく、過去に実施した月経痛患者向けの予備的研究では、参加患者より月経困難症の大幅な改善や子宮内膜症の病状の改善をみとめたことから、大規模なプラセボ対照研究により子宮内膜症による疼痛および子宮内膜症の改善効果が検証でき、製品化・保険収載等に進めば、患者にとっての利益が非常に大きいと判断し、この度の臨床研究を計画した。

---

[1] 難治性希少部位子宮内膜症の集学的治療のための分類・診断・治療ガイドライン

[2] 試験機器概要書

[3] A Multicenter, Prospective, Randomized, Placebo-Controlled, Double-Blind Study of a Novel Pain Management Device, AT-02, in Patients with Fibromyalgia, Pain Medicine, Volume 21, Issue 2, February 2020, Pages 326–332, Published: 26 November 2019

1.2. これまでに実施されてきた標準治療の経緯及び内容[4]

政府統計によれば、平成 26 年度 10 月の推計患者数は 4,700 人であるとされている。これは昭和 59 年と比較すると約 2 倍の数値になり、平成 26 年度の総患者数は 54,000 人だとされている。なお、「日本子宮内膜症啓発会議」の見解によれば、2013 年時点での子宮内膜症患者数は推計 260 万人以上であるとされており、政府統計との乖離の原因は「子宮内膜症の症状」によるものである。

子宮内膜症は、生理周期に伴う生理痛の悪化や不妊といった症状であるが、症状は劇的に変化するわけではなく、「生理痛が重くなった」という軽い自覚が中心となるため、子宮内膜症を自覚して婦人科を受診する女性が少ないことによる。

先ほどの政府統計によると、平成に入ってから子宮内膜症の患者数は増加傾向にあり、その理由は「女性のあり方」が、ここ数十年で大きく変化したことにある。

子宮内膜症の罹患率が上昇傾向で推移している最大の原因は「女性の社会進出」と、それに伴う「女性のライフスタイルの変化」であり、1990 年代から、女性の社会進出が顕著になり、それまでの「女性は家庭に入る」という考えが少しずつ見直された。女性の多くが会社等で働くようになり、ライフスタイルもそれに応じて変化している。

その最大の影響は「晩婚化」と「晩産化」である。社会進出するようになったことで女性の結婚および初婚の年齢が一昔前より高くなり、社会進出による女性に対するストレスの増加も危惧されている。

晩婚化と晩産化が子宮内膜症に及ぼす影響は「月経回数の増加」であり、子宮内膜症の詳しい発症原因は特定されていないが、月経が深く関係し、月経を経るごとに症状が悪化するということは判明している。そのため薬物療法として偽閉経療法や偽妊娠療法がとられることがあるが、晩産化によって若年層の月経回数が増加したことによって月経に関係する病気の発症リスクが高まった。

子宮内膜症の治療としては、大きく分けて薬による治療と手術による治療があり、症状の種類や重症度はもちろん、年齢、妊娠の希望などを総合的に判断して最適な治療法が選択される。

痛みに対してはまず、鎮痛剤を使用され、効果が得られない時はホルモン量の少ないピル（低用量エストロゲン・プロゲスチン配合薬／低用量ピル）が用いられる。視床下部ホルモンである GnRH アナログ製剤（アゴニスト・アンタゴニスト）や黄体ホルモン剤などが用いられることもあり、女性ホルモンの分泌を抑えたり直接病巣に作用させたりして症状を緩和させる。

卵巣の内膜症性のう胞（チョコレートのう胞）などの病巣部がはっきりしている場合は、手術を考慮する。妊娠を望んでいる場合は、病巣部のみを切除して子宮や卵巣の正常部分を残す手術を選択する。妊娠を望まない場合には、病巣のみの摘出に加えて、子宮、卵巣および卵管などを摘出することもある。

1.3. 現在の標準治療の内容及び治療成績（臨床試験成績を含む）[5]

子宮内膜症治療の目的は以下の 3 点である。

①月経困難症・慢性骨盤痛などの疼痛の緩和
②不妊症に対する妊孕性改善
③卵巣チョコレート嚢胞の破裂、感染、癌化の予防

[4] 政府統計による子宮内膜症患者数
http://www.mhlw.go.jp/toukei/saikin/hw/kanja/10syoubyo/dl/h26syobyo.pdf
[5] 公益社団法人　日本産婦人科学会　研修ノート（4）子宮内膜症への対応

①対症療法：NSAIDs をはじめとする鎮痛薬や漢方薬。
②内分泌療法：LEP 製剤、ジエノゲスト、ジドロゲステロン、LNG-IUS、GnRH アナログなど。
③手術療法：卵巣チョコレート嚢胞摘出術、付属器摘出術、子宮内膜症病巣摘出、癒着剝離術などがある。近年は大部分が腹腔鏡手術で行われている。

子宮内膜症治療では疼痛による QOL の低下を改善することが主体となるが、若年女性では将来の妊孕性温存や、高年女性では卵巣チョコレート嚢胞の悪性化予防や早期発見が重要であるため、ライフステージ全体を見据えて治療の選択を行う必要がある。

子宮内膜症に対する薬物療法の基本的な考え方。

①疼痛緩和<br>②不妊の原因となる病巣の改善<br>③病巣の縮小ないし消失による手術効果の向上<br>④再発・再燃に対して病巣の進行を遅らせる、あるいは再発の予防

1 ） 薬物療法の分類
・対症療法と内分泌療法に大別される。

対症療法	内分泌療法
非ステロイド性消炎鎮痛薬（NSAIDs）<br>漢方薬	ジエノゲスト<br>低用量エストロゲン・プロゲスチン配合薬（LEP）<br>GnRH アナログ製剤（アゴニスト・アンタゴニスト）<br>ダナゾール<br>レボノルゲストレル放出子宮内システム（LNG-IUS）<br>アロマターゼ阻害薬＊<br><br>＊保険未収載

・対症療法

薬剤	効果
NSAIDs	月経痛などの一時的な疼痛を緩和することに効果がある。
漢方薬	弱い疼痛緩和効果があり、子宮内膜症に起因した気分不快などを緩和し、内分泌療法に伴う低エストロゲン症状を軽減する効果がある。
内分泌療法	病巣に働きかける薬物療法は、現時点では内分泌製剤だけである。<br>作用機序は、排卵を抑制することにより、卵巣に由来する内因性エストロゲン分泌を低下させて子宮内膜症病巣を退縮させることと、子宮内膜症病巣局所に直接作用して退縮させることがある。LEP 製剤、ジエノゲストを第 1 選択とし、GnRH アナログ製剤、ダナゾールを第 2 選択として投与する。疼痛抑制作用は GnRH アナログ製剤が最も強い。
薬物療法	強い癒着を伴う深部子宮内膜症には効果が乏しいことが多く、大きい卵巣チョコレート嚢胞の縮小効果や不妊の改善効果はあまり期待できないことから、基本的に腹腔鏡手術や ART を選択する

2 ） 長期維持管理のための内分泌療法
・短期間の内分泌療法では、投与を中止した場合速やかに再発することも多い。

・疼痛の抑制あるいは術後の疼痛や病巣の再燃を長期間抑制するためには、内分泌療法を長期間継続する工夫が必要である。
・GnRH アナログ製剤先行投与法は、疼痛抑制効果は強いが長期には投与できない。GnRH アゴニストの 4～6 カ月投与に引き続き、低用量の LEP 製剤、ジエノゲストあるいはダナゾールを長期間にわたって投与し続けることで、GnRH アナログ製剤で得られた疼痛抑制効果を長期に維持することが可能となる。

3 ）内分泌療法と副作用
① LEP 製剤と血栓症
LEP 製剤により静脈血栓塞栓症（VTE : venous thromboembolism）リスクが増加するとされる。実際の年間発症率は 1 万人あたり 9 人程度で稀であるが、VTE 予防の観点からは、エストロゲン含量の少ない薬剤の使用が望ましい。術前 4 週以内、術後 2 週以内、産後 4 週以内および長期間安静状態の患者には禁忌である。VTE 発症の前兆としては、以下の症状 : ACHES（A : Abdominal pain ; 腹痛、C : Chest pain ; 胸痛・突然の息切れ、H : Headache ; 激しい頭痛、E : Eye/speech problem ; 急性視力障害・構語障害、S：Severe leg pain ; 下肢の疼痛・浮腫）に注意する。

②ジエノゲストと不正子宮出血
ジエノゲストは、病巣への直接作用を有し、慢性深部痛に対する効果も高い。また、肝機能、脂質代謝や凝固能への影響が少なく、血栓症のリスクなどから閉経周辺期や肥満事例などの LEP 製剤が使いにくい場合にも使用できる。本剤では、GnRH アゴニストでみられるエストロゲン欠乏症状が少ない。副作用として、使用後数カ月間の不正子宮出血が挙げられるが、継続的投与により出現頻度は減少する。

4）手術療法
・子宮内膜症の手術は病巣の焼灼、癒着剥離からチョコレート囊胞や深部病巣の摘出など多岐にわたり、患者の症状や年齢、悪性所見の有無などで適応や術式を決定していく。
・子宮、卵巣の解剖学的位置関係は病巣の癒着や繊維化により変化し、その程度により手術の難易度が大きく変わる。術者はいかなる状況にも対応できるよう剥離や結紮縫合などの手技を研鑽するとともに、消化器外科や泌尿器科とのスムーズな連携体制を構築しておく必要がある。
・いかなる手術も再発のリスクは避けられないことから、術後の長期フォローアップの必要性や積極的なホルモン製剤の予防投与についても、術前から患者に十分に説明し同意を得ておくことが大切である。

1.4. 当該臨床研究の必要性につながる、現在の標準治療の課題、不明点等 [6]

本研究で用いる試験機器（AT-04）は、人体にほぼ無害な 2kHz 及び 83.3MHz の微弱な交番磁界（磁界エネルギーは地磁気の約 1/3 程度）を同時発生させる超低侵襲的治療機器であり、患部にあてることにより痛みの緩和・治療効果を実現するものである。
本機器が疼痛改善効果を発揮する機序としては、過去のマウス等を用いた非臨床試験の結果より、神経成長因子の調整、末梢における炎症性サイトカインの抑制および下行抑制系の賦活化であると考えられており、上述のような本症の病理学的背景に鑑みると、本機器が子宮内膜症の病巣やそれに起因する疼痛に有効な可能性は十分期待できる。実際、本機器の臨床試験では、すでに線維筋痛症 1)、腰痛症 2)などで有効性かつ有害事象が発生しないという安全性を示しているだ

[6] 試験機器概要書

けでなく、過去に実施した月経痛患者向けの予備的研究では、参加患者より月経困難症の大幅な改善や子宮内膜症の病状の改善が報告されていることから、大規模なシャム機対照研究により子宮内膜症による疼痛および子宮内膜症の改善効果が検証でき、適用追加による保険収載等に進めば、患者にとっての利益が非常に大きいと判断し、この度の臨床研究を計画した。

1.5. 当該臨床研究に用いる医薬品等に関する情報[7]

交番磁界治療器

（1）類別名称：理学診療用器具

（2）クラス分類：管理医療機器・特定保守管理医療機器

（3）販売名：エイト

（4）承認・認証・届出番号：承認番号：30400BZX00015000

（5）使用目的又は効果： 本品は、2 種類の交番磁界を経皮的に照射し、神経を刺激することで疼痛を緩和させるために用いられる。

（6）使用方法： 添付文書又は取扱説明書参照

（7）製造販売業者等：株式会社Ｐ・マインド

（8）その他：添付文書参照又は取扱説明書参照

2. 試験の目的

本試験の目的は、子宮内膜症患者において、AT-04 照射群とシャム機 S-02（非照射）群との NRS 値の照射前及び照射 16 週後における変化量を群間で比較し、AT-04 照射による有効性を検討する。

また、不具合、有害事象の発現率を群間で比較し、AT-04 照射の安全性についても検討する。

本試験の妥当性について、当該試験はシャム機対照試験であり、実機の有効性を比較するために、シャム機に割り付けられた患者には試験開始後 16 週間はシャム機を使用してもらうが、16 週以降の 4 週間は全被験者に実機を使用する倫理的に許容されるデザインとした。

3. 対象疾患

子宮内膜症

3.1. 選択基準

以下の選択基準を全て満たす者を対象とする。

（1）同意取得時に、18 歳以上の患者

（2）閉経前の女性患者

（3）子宮内膜症患者（以下のいずれかに該当する者とする。なお、術後再発した場合は、再度、以下のいずれかで診断されていること）

①治療開始前の 5 年以内に実施した開腹又は腹腔鏡検査により子宮内膜症と診断された患者

②治療開始前の 1 年以内に実施した MRI または治療開始前に実施した超音波検査（経腟、経腹又は経直腸）により卵巣チョコレート嚢胞等画像上検出される子宮内膜症を認める患者

③治療開始前に実施した内診・直腸診によりダグラス窩の硬結、子宮可動性の制限または骨

[7] エイト　添付文書

盤の圧痛のいずれかを認め、臨床子宮内膜症と診断される患者
（4）子宮内膜症に由来すると考えられる月経困難症または骨盤痛を有し、治療開始前にて B&B 評価スケールで少なくとも一方が中等度以上であると研究責任（分担）医師が判断した患者
（5）同意取得前の 28 日間において、子宮内膜症による痛み NRS（Numeric Rating Scale）スコアの平均が 4 以上である患者
（6）同意取得前の 28 日間において、子宮内膜症に対する治療の新たな開始、もしくは治療内容（薬の処方内容・用法・用量を含む）の変更がない患者
（7）同意取得前の 28 日間において、子宮内膜症の急性憎悪を疑う所見がないと研究責任（分担）医師が判断した患者
（8）本人の自由意思により、研究の参加について文書による同意を得られている患者

【設定の根拠】
（1）試験機器の有効性が期待される対象として設定した
（2）対象疾患の主対象として設定した
（3）対象疾患の主対象として設定した
（4）試験機器の有効性を適切に評価するために設定した
（5）試験機器の有効性を適切に評価するために設定した
（6）試験機器の有効性を適切に評価するために設定した
（7）試験機器の有効性を適切に評価するために設定した
（8）臨床研究法に遵守するために設定した

3.2. 除外基準

以下の除外基準に 一つでも該当する者は対象から除外する。
（1）以下の医薬品等を同意取得の 8 週前以内に使用した患者
・臨床試験薬または治験薬
・GnRH アナログ製剤、ダナゾール及びアロマターゼ阻害剤、選択的エストロゲン受容体モジュレーター製剤
（2）試験機器を含む交番磁界治療機器を過去に使用したことのある患者
（3）NSAIDs を定期的に服用している患者
（4）卵巣チョコレート嚢胞が 10 cm 以上、かつ年齢が 40 歳以上の患者
（5）両側卵巣摘出術の既往がある患者
（6）著しい不正子宮出血、原因不明の不正子宮出血の合併があると研究責任（分担）医師が判断した患者
（7）試験期間中に新たに治療を要すると研究責任（分担）医師が判断する子宮筋腫を合併している患者
（8）過敏性腸症候群および・または重度の間質性膀胱炎による下腹部痛を有する患者
（9）重度の肝障害、黄疸、腎障害、心血管系疾患、内分泌系疾患、代謝疾患、肺疾患、胃腸疾患、神経疾患、泌尿器疾患、免疫疾患、並びに精神疾患（特にうつ様症状）およびそれに起因する自殺企図等の既往または合併がある患者
（１０）人工心肺及びペースメーカ等の生命維持用医用電気機器を使用している患者
（１１）心電計等の装着型医用電気機器を使用している患者
（１２）他の医薬品や医療機器の治験や臨床試験に参加している患者
（１３）治療のために入院の必要がある患者

【設定根拠】

(1)-(6)：本試験の有効性の評価に影響を与えると考えられるため、設定した。
(7)-(11)：対象患者の安全性を考慮し、設定した。

4. 被験者の同意

4.1. 同意文書及びその他の説明文書の作成並びに改訂

研究責任医師は、被験者から試験参加の同意を得るために用いる同意文書及びその他の説明文書を可能な限り平易な表現で作成する。また、同意文書及びその他の説明文書を改訂する必要があると認めた場合は、これらを改訂する。

研究責任医師は、作成又は改訂された同意文書及びその他の説明文書を臨床研究審査委員会に提出し、その承認を得る。

4.2. 同意取得の時期と方法

1) 同意の取得

研究責任医師又は研究分担医師は、同意文書及びその他の説明文書を被験者に手渡し、「4.3 被験者に対する説明事項」に示す内容について十分な説明を行う。また、必要な場合には、研究協力者も被験者に補足的な説明を行う。被験者が試験の内容を良く理解したことを確認した上で、試験開始前(スクリーニング)検査を実施するまでに文書で自由意思による同意を取得する。

2) 説明時の被験者への対応

研究責任医師又は研究分担医師は、同意を得る前に被験者が質問をする機会と当該試験に参加するか否かを判断するのに十分な時間を与え、被験者の質問に対しては、被験者が満足するように回答する。

3) 同意書への記入方法および説明文書の交付

被験者及び被験者の同意に際しては、説明を行った研究責任医師又は研究分担医師が記名捺印又は署名し、説明した日付を記入する。被験者は同意書に署名し、同意した日付を記入する。なお、研究協力者が補足的な説明を行った場合は、当該研究協力者も記名捺印又は署名し、説明した日付を記入する。同意を得た後、説明文書及び同意書の写しを被験者に交付する。

4) 説明文書改訂時

研究責任医師又は研究分担医師は、被験者の同意に関連し得る新たな情報の入手などにより同意文書及びその他の説明文書を改訂した場合、被験者に対して改訂された同意文書及びその他の説明文書を用いて改めて説明し、研究への参加継続について文書で同意を取得する。

4.3. 被験者に対する説明事項

研究責任医師が作成する説明文書には、以下の事項を記載する。

1) 本臨床研究の名称、本臨床研究の実施について研究実施医療機関の管理者の承認を受けている旨及び厚生労働大臣に実施計画を提出している旨
2) 研究実施医療機関の名称並びに研究責任医師の氏名及び職名（研究を総括する医師の氏名及び職名、他の研究実施医療機関の名称並びに当該研究実施医療機関の研究責任医師の氏名及び職

名を含む）
3) 本臨床研究の対象者として選定された理由
4) 本臨床研究の実施により予期される利益及び不利益
5) 本臨床研究への参加を拒否することは任意である旨
6) 同意の撤回に関する事項
7) 本臨床研究への参加を拒否すること又は同意を撤回することにより不利益な取扱いを受けない旨
8) 本臨床研究に関する情報公開の方法
9) 本臨床研究の対象者の求めに応じて、研究計画書その他の本臨床研究の実施に関する資料を入手又は閲覧できる旨及びその入手又は閲覧の方法
10) 本臨床研究の対象者の個人情報の保護に関する事項
11) 試料等の保管及び廃棄の方法
12) 本臨床研究に対する利益相反管理に関する状況
13) 苦情及び問合せへの対応に関する体制
14) 本臨床研究の実施に係る費用に関する事項
15) 他の治療法の有無及び内容並びに他の治療法により予期される利益及び不利益との比較
16) 本臨床研究の実施による健康被害に対する補償及び医療の提供に関する事項
17) 本臨床研究の審査意見業務を行う認定臨床研究審査委員会における審査事項その他本臨床研究に係る認定臨床研究審査委員会に関する事項
18) その他本臨床研究の実施に関し必要な事項

「その他当該臨床研究に関し必要な事項」は、次に掲げる事項を含むこと。

① 当該臨床研究の参加を中止する場合の条件及び理由
② 臨床研究への参加の継続について臨床研究の対象者の意思に影響を与える可能性のある情報が得られたときは、速やかに説明し、参加の継続の意思を再度確認する旨
③ 規則第 21 条第 1 項第 1 号[※1]及び第 2 号[※2]に定める医薬品等製造販売業者等の当該臨床研究に対する関与の有無とその内容：
  ※1 当該研究責任医師が実施する臨床研究に対する医薬品等製造販売業者等による研究資金等の提供その他の関与
  ※2 当該臨床研究に従事する者及び研究計画書に記載されている者であって、当該臨床研究を実施することによって利益を得ることが明白な者に対する、当該臨床研究に用いる医薬品等の製造販売をし、又はしようとする医薬品等製造販売業者等による寄付金、原稿執筆及び講演その他の業務に対する報酬の提供その他の関与
④ モニタリング、監査等において認定臨床研究審査委員会、厚生労働省等が臨床研究に係る資料を閲覧することがある旨及びその際、個人情報が適正に利用され、同意文書に署名することで当該閲覧を認めたことになる旨
⑤ 研究責任医師又は研究分担医師の氏名と連絡先
⑥ 臨床研究の対象者が守るべき事項

5. 試験のデザイン

5.1. 評価項目

1）主要評価項目

治療開始前のベースラインと比較した、二重盲検期終了時（16 週後）における子宮内膜症関連疼痛の各来院時に収集した思い出しによる Numeric Rating Scale (NRS)スコアの変化量

2）副次評価項目

【有効性の副次評価項目】

- 治療開始前のベースラインと比較した、4,8,12,20 週後における子宮内膜症関連疼痛の各来院時に収集した思い出しによる Numeric Rating Scale (NRS)スコアの変化量
- 治療開始前のベースラインと比較した、治療期間中における月経痛および月経期間の子宮内膜症骨盤痛の ePRO において収集した毎日の NRS スコアの変化量
- 治療開始前のベースラインと比較した、治療期間中における月経期間以外の子宮内膜症骨盤痛の ePRO において収集した毎日の NRS スコアの変化量
- 治療開始前のベースラインと比較した、4,8,12,16,20 週後の Biberoglu & Behrman (B&B) スコアの変化量
- 治療開始前のベースラインと比較した、4,8,12,16,20 週後の Endometriosis Health Profile-30 (EHP-30) スコアの変化量
- 治療開始前のベースラインと比較した、4,8,12,16,20 週後の健康状態包括的尺度 (EQ-5D) の変化量
- 治療開始前のベースラインと比較した、二重盲検期間終了後（16 週後）の卵巣チョコレート嚢胞の体積の変化量

【安全性の副次評価項目】

試験機器の不具合及び有害事象の発現頻度・割合

5.2. 試験デザインの概略

本試験は、無作為化比較、並行群間比較、プラセボ対照、二重盲検、多施設共同試験である。

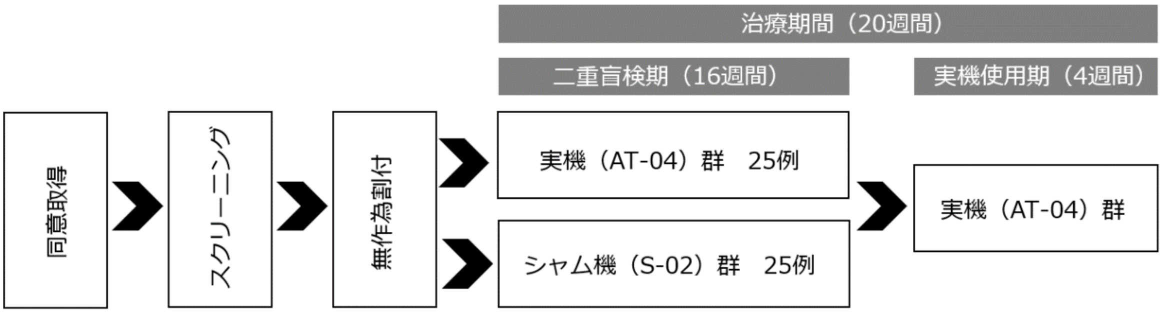

試験デザインの概略

5.3. 試験の方法

本試験は同意取得後、スクリーニング検査にて適格性を確認し、データセンターにおいて中央登録及びバイアスを最小化するための無作為化割付を 1 : 1 で行う。（詳細は「5.5 症例登録・割付

方法」に記載）

実機治療群には、AT-04 を 16 週間施術する。また、対照群にはシャム機である S-02 を 16 週間施術する。

16 週の来院時に、被験者に機器を持参してもらい、新しい機器（全て実機）に交換する。実機使用期間としてその後 4 週間施術する。16 週以降、実機交換後の盲検性を担保するため、本試験への参加の同意を取得する際に、試験機器によって使用感が変わる可能性があることを説明する。

なお、パッドの装着部位は、子宮・卵巣付近を含む下腹部に最低 2 箇所、他に疼痛部がある場合は追加としてその部位に 2 箇所（最大 4 箇所）貼付する。

5.4. 目標被験者数と試験実施期間

目標被験者数 : 50 例（各群 25 例）

試験実施期間： jRCT 公表日 - 2025 年 3 月 31 日

症例登録期間： jRCT 公表日 - 2024 年 3 月 31 日

5.5. 症例登録・割付方法

5.5.1. 施設登録

1) 施設登録および症例登録は、データセンター（千葉大学医学部附属病院）における中央登録制とする。
2) データセンターは、jRCT にて各実施医療機関で当該特定臨床研究に対する管理者の許可の有無が「あり」になっていることを確認し、施設登録を行う。
3) データセンターは施設登録を行った後、施設登録完了通知書の写しを研究責任医師に送付する。

5.5.2. 症例登録・割付方法

1) 登録は、同意取得及びスクリーニング検査後、治療開始日（Day1）までに行うこととする。
2) 研究責任医師または研究分担医師は、文書による同意を取得した後、スクリーニング検査を実施し、選択基準・除外基準を満たしているかを確認する。
3) 研究責任医師または研究分担医師は、症例登録票を作成し、必要事項が全て記入されている事を確認の上、データセンターに FAX する。
4) データセンターは被験者の適格性を確認し、適格／不適格の症例登録結果及び割付結果を研究責任医師または研究分担医師に FAX により伝達する。
5) 研究責任医師または研究分担医師は、「適格」と判定された被験者に対して、割付結果に従いプロトコル治療を開始する。

＊ 誤登録・重複登録が判明した際には速やかにデータセンターに連絡すること。

5.5.3. 割付調整因子

無作為割り付けに際しては、①試験施設、②各群内の LEP・プロゲスチン治療有無を調整因子とする最小化法を用いる。

5.5.4. 症例登録先

千葉大学医学部附属病院　データセンター

受付時間：午前 9 時～午後 5 時（土日、祝日、年末年始を除く）

※受付時間外に FAX を受信した場合、翌稼働日の受付とする

TEL：043-221-7171（内線：6593）

FAX：043-226-2644

5.6. 不適格となった被験者の取り扱い

登録において、割付前に不適格などの何らかの理由で割付が行われなかった場合は、登録されなかった被験者となり、試験の登録症例には含めない。研究責任医師又は研究分担医師は、当該被験者に本試験への登録が不可である旨を説明するとともに記録として残す。

5.7. 試験機器の施術スケジュールおよび機器の使用方法

実機（AT-04）、シャム機（S-02）ともに連日 1 回当たり 30 分、1 日 2 回（60 分）以上、最大 4 回まで（最大 2 時間）、下腹部 2 箇所に使用する。使用時の姿勢は問わない。なお、下記の図に示した基本貼付部位以外に痛みを感じる場合は、当該部位に追加で 2 箇所に使用出来る。

パッドは、専用の両面テープ、サージカルテープで固定するか、サポーター等を用い固定する。

基本貼付部位（●）

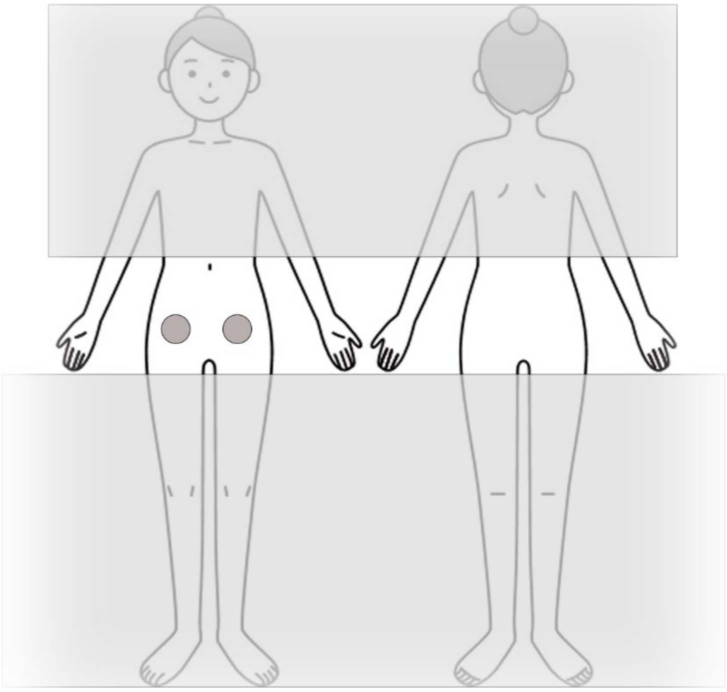

5.8. 休止（中断）及び再開の基準

研究責任医師又は研究分担医師が試験機器の使用継続を不適当と認めた場合、研究責任医師又は研究分担医師は機器の使用を一旦休止（中断）する。ただし、検査スケジュールに沿った検査・評価は継続して実施する。

なお、研究責任医師又は研究分担医師が、被験者の状態が改善し機器使用の再開が可能と判断した場合は再開する。

5.9. 機器使用中止基準

試験機器の 1 日あたりの使用回数もしくは使用パッド数を制限しても有害事象が発現し、かつ研究責任医師又は研究分担医師が試験機器の使用中止を必要と認めた場合、研究責任医師又は研究分担医師は試験機器の使用を中止する。ただし、検査スケジュールに沿った検査・評価は継続

して実施する。

5.9.1. 被験者の妊娠

被験者の妊娠が判明した場合は、研究責任医師又は研究分担医師が機器の安全性に関して被験者に対し十分に説明した上で、被験者の同意が得られれば使用を継続し、安全性評価を行うために検査スケジュールに沿った検査のみ継続する。

5.10. 個々の症例の中止基準

以下の基準に該当した場合、研究責任医師又は研究分担医師は試験機器使用を中止し、さらに被験者を試験中止とする。その場合、中止時の検査・評価を実施する。

1) 試験機器の使用継続が困難な有害事象が発現し、かつ研究責任医師又は研究分担医師が被験者の試験中止を必要と認めた場合。
2) 被験者が同意を撤回した場合。
3) 被験者から中止の申し出があった場合。
4) その他、研究責任医師又は研究分担医師が被験者の試験継続が不可能と判断した場合。
5) 研究代表医師等により試験全体の中止が決定された場合。

【各中止基準の設定根拠】<br>被験者の安全性確保を目的とした。

5.11. 併用薬及び併用療法

5.11.1. 併用制限薬

1) 標準的治療：原疾患および合併症に付随する症状に対する標準的治療として同意取得時に投与中の薬剤は継続する。原則として、試験参加中（同意取得時～試験終了又は中止）は、薬剤の変更、用法・用量の変更は行わないこととするが、変更を行った症例については、その取り扱いについて試験終了後に検討する。
2) アスピリン：虚血性心疾患あるいは虚血性脳血管障害に対して用いる抗血小板作用を目的とする場合、325mg/日以下の用量での使用を可能とする。また、やむを得ず疼痛緩和目的で使用する場合に限り、使用を可能とする。
3) アセトアミノフェン：解熱目的かあるいはやむを得ず疼痛緩和目的で使用する場合に限り、1日 1,500mg までの使用を可能とする。
4) アスピリン、アセトアミノフェン以外の NSAIDs：やむを得ず疼痛緩和目的で使用する場合に限り、使用を可能とする。

5.11.2. 併用禁止薬

以下の薬剤については、試験参加中（同意取得時～試験終了又は中止）は使用することができない。また、試験開始前より使用していた場合は継続不可とする。

子宮内膜症治療剤
• GnRH アナログ製剤<br>• ダナゾール<br>• アロマターゼ阻害剤<br>• 選択的エストロゲン受容体作動薬

疼痛緩和薬
• 麻薬性及び非麻薬性鎮痛薬（トラマドール等）

- 疼痛緩和目的で使用される局所麻酔薬

5.12. 併用禁止療法

以下の治療法については、試験参加中（同意取得時～試験終了又は中止）は行うことができない。また、試験開始前より行っていた場合は継続不可とする。

1) 疼痛緩和目的の治療（いわゆる民間療法など）
2) 経皮電気神経刺激装置（TENS）
3) トリガーポイント注射
4) 神経ブロック

5.13. 試験終了後の対応

本試験が終了した後に受ける治療は規定しない。

研究責任医師又は研究分担医師は、被験者が試験終了後においても試験の結果により得られた最善の予防、診断及び治療を受けることができるよう努める。

6. 試験機器

6.1. 試験機器の概要 [8]

本試験で使用する試験機器は以下のとおりである。試験機器の詳細及び取り扱いに関しては、添付文書参照又は取扱説明書を参照すること。

類別名称	理学診療用器具
クラス分類	管理医療機器・特定保守管理医療機器
商品名	エイト
保管方法	1. 保管条件<br>周囲温度：-20～60℃<br>相対湿度：30～96%（結露なきこと）<br>2 .動作保証温度<br>周囲温度:0～35℃<br>3. 耐用期間<br>5 年［自己認証（当社データ）による］
製造販売元	株式会社Ｐ・マインド
使用方法	試験機器の導子を下腹部に当て、1 回あたり 30 分、1 日 2 回以上（最大 2 時間以内）使用する。
安全性情報	先行治験では、機器の不具合の有無と発生頻度、重篤な有害事象を含む有害事象の有無と発現頻度について評価した。その結果、機器の不具合、並びに重篤な有害事象は認められなかった。有害事象は、安全性解析対象集団（30 例）において 9 例 9 件（30.0 %）に発現した。全 9 例中、機器との因果関係が否定できない有害事象は、一般・全身障害及び投与部位の状態（医療機器使用部位反応）が 4 例 4 件（13.3 %）であった。当該有害事象は専用粘着シートによるかぶれ・掻痒感であった。
シャム機	本試験で使用するシャム機は、P・マインド社が製造するシャム機 S-02 を利用する。交番磁界治療器 AT-04 と外観や操作は全く同じであるが、導子のコイルに電流が流れても磁界が発生しない構造になっている。そのため、使用者は AT-04 と区別することが不可能である。
機器の外観	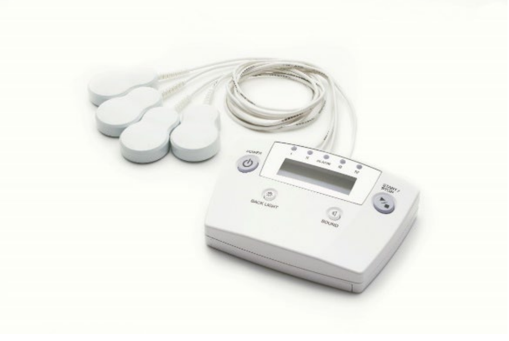<br>（実機・シャム機共通）

6.2. 試験機器の管理方法

当該試験機器は、試験機器の管理に関する手順書に従って管理を行う。

[8] 試験機器概要書、エイト添付文書

7. 観察・検査・評価項目、方法及び実施時期

7.1. 実施スケジュールと手順

観察・検査・評価の実施スケジュールを以下のスケジュール表に示す。研究責任医師又は研究分担医師は、スケジュールに従って観察・検査・評価を実施する。

スケジュール表

		スクリーニング	二重盲検期					実機使用期	中止時
			Day1 ※1	4 週後	8 週後	12 週後	16 週後	20 週後	
許容範囲		-35	-	±7	±7	±7	±7	±7	-
同意取得 ※2		●							
被験者背景	年齢	●							
	身長	●							
	体重・BMI	●					●	●	●
	バイタルサイン（血圧・体温）	●		●	●	●	●	●	●
	既往歴・合併症	●							
	前治療	●							
	子宮内膜症主要症状を含む身体所見	●							
	自他覚症状	●		●	●	●	●	●	●
	超音波検査	●					●		●
評価項目（疼痛）	NRS スコア ※3	●	○	●	●	●	●	●	●
	NRS スコア（月経期間）	●		●※4	●※4	●※4	●※4	●※4	●※4
	NRS スコア（月経期間以外）	●		●※4	●※4	●※4	●※4	●※4	●※4
副次評価（子宮内膜症）	B&B スコア	●	○	●	●	●	●	●	●
	EHP-30 スコア		○	●	●	●	●	●	●
	EQ-5D スコア		○	●	●	●	●	●	●
有害事象		——	——	——	——	——	——	→	●
不具合			——	——	——	——	——	→	●
併用薬・併用療法		——	——	——	——	——	——	→	●
試験機器	登録・割付	●							
	交換						●		
患者日誌 ※5	月経の有無	——	——	——	——	——	——	→	●
	機器使用回数	——	——	——	——	——	——	→	●
	パッド使用枚数	——	——	——	——	——	——	→	●
	NRS スコア	——	——	——	——	——	——	→	●
	鎮痛剤使用有無	——	——	——	——	——	——	→	●
	不具合・有害事象の有無	——	——	——	——	——	——	→	●
中止理由									●

各来院時に実施する検査、評価項目を●で示している。

※1 Day1 の規定検査（○）は、許容範囲を-14 日とする。試験機器による治療は Day1 より開始する。

なお、Day1 より前に規定検査を実施し、かつ試験機器の交付を受けた被験者は、Day1 当日の来院は不要。

※2 被験者からの文書同意取得は Day-28 までに実施する。

※3 来院ごとに、過去 4 週間の疼痛の程度を思い出し、評価する。

※4 患者報告アウトカムの電子データ（Electronic Patient Reported Outcome）システムにて収集したデータを用いて評価する。

※5 患者日誌は同意取得日より記録を開始する。Day1 前日までは紙の患者日誌への記録、Day1 以降は ePRO による記録とする。

7.1.1. スクリーニング

被験者からの文書同意取得は Day-35～Day-28 の間に実施する。同意取得後、スクリーニングを行う。研究責任医師又は研究分担医師は以下のスクリーニング項目を確認し、選択基準を満たし、除外基準に抵触しない患者を被験者とする。検査項目は以下に記載の通りとする。

① 被験者背景の調査（同意取得日、被験者識別コード、年齢、身長（cm）、体重（kg）、BMI、既往歴・合併症、前治療、子宮内膜症主要症状を含む身体所見、過去の経妊回数、過去の経産回数）
② バイタルサイン（収縮期血圧/拡張期血圧・体温）
③ 自他覚症状（疼痛関連症状）
④ NRS スコア（来院前 4 週間の平均値）※
⑤ B&B スコア
⑥ 超音波検査（卵巣チョコレート嚢胞の有無、病変の部位、卵巣チョコレート嚢胞の個数、卵巣病変の場合はそれぞれの嚢胞の最大の直径とそれに直交する直径、直径から算出される体積（cm^3））
⑦ 有害事象の確認
⑧ 併用薬、併用療法

※NRS スコアは来院前 4 週間の子宮内膜症骨盤痛を被験者に思い出してもらうことによって評価する。

7.1.2. 観察・検査・評価項目

来院ごとの観察、検査及び評価項目を以下に記す。なお、試験機器の使用開始日（Day1）が同意取得から 35 日を超えた場合は、再度、文書同意を取得し、スクリーニング検査を実施することとする。

Day1 から被験者が試験機器を使用できるよう、「**5.5.2. 症例登録・割付**」に従い、事前に症例登録及び割付を実施する。

7.1.2.1. Day1（0 週）

Day1 から試験機器による治療を開始する。Day1 の検査項目については許容範囲を-14 日とする。Day1 の検査項目を実施済みかつ試験機器の提供を Day1 以前に受けた被験者は、Day1 の来院は不要とする。

① NRS スコア（来院前 4 週間の平均値）※
② B&B スコア、EHP-30 スコア、EQ-5D スコア

※NRS スコアは来院前 4 週間の子宮内膜症骨盤痛を被験者に思い出してもらうことによって評価する。

7.1.2.2. 4 週後

① バイタルサイン（収縮期血圧/拡張期血圧・体温）
② 自他覚症状（疼痛関連症状）
③ NRS スコア（0 週から 4 週までの平均値）※
④ NRS スコア（0 週から 4 週における月経期間の最大値及び平均値）
⑤ NRS スコア（0 週から 4 週における月経期間以外の最大値及び平均値）
⑥ B&B スコア、EHP-30 スコア、EQ-5D スコア
⑦ 有害事象、不具合の確認

⑧ 併用薬、併用療法

※NRS スコアは来院前 4 週間の子宮内膜症骨盤痛を被験者に思い出してもらうことによって評価する。

7.1.2.3. 8 週後

① バイタルサイン（収縮期血圧/拡張期血圧・体温）
② 自他覚症状（疼痛関連症状）
③ NRS スコア（4 週から 8 週までの平均値）※
④ NRS スコア（4 週から 8 週における月経期間の最大値及び平均値）
⑤ NRS スコア（4 週から 8 週における月経期間以外の最大値及び平均値）
⑥ B&B スコア、EHP-30 スコア、EQ-5D スコア
⑦ 有害事象、不具合の確認
⑧ 併用薬、併用療法

※NRS スコアは来院前 4 週間の子宮内膜症骨盤痛を被験者に思い出してもらうことによって評価する。

7.1.2.4. 12 週後

① バイタルサイン（収縮期血圧/拡張期血圧・体温）
② 自他覚症状（疼痛関連症状）
③ NRS スコア（8 週から 12 週までの平均値）※
④ NRS スコア（8 週から 12 週における月経期間の最大値及び平均値）
⑤ NRS スコア（8 週から 12 週における月経期間以外の最大値及び平均値）
⑥ B&B スコア、EHP-30 スコア、EQ-5D スコア
⑦ 有害事象、不具合の確認
⑧ 併用薬、併用療法

※NRS スコアは来院前 4 週間の子宮内膜症骨盤痛を被験者に思い出してもらうことによって評価する。

7.1.2.5. 16 週後

① 体重、BMI
② バイタルサイン（収縮期血圧/拡張期血圧・体温）
③ 自他覚症状（疼痛関連症状）
④ NRS スコア（12 週から 16 週までの平均値）※
⑤ NRS スコア（12 週から 16 週における月経期間の最大値及び平均値）
⑥ NRS スコア（12 週から 16 週における月経期間以外の最大値及び平均値）
⑦ B&B スコア、EHP-30 スコア、EQ-5D スコア
⑧ 超音波検査（卵巣チョコレート嚢胞の有無、病変の部位、卵巣チョコレート嚢胞の個数、卵巣病変の場合はそれぞれの嚢胞の最大の直径とそれに直交する直径、直径から算出される体積（cm^3））
⑨ 有害事象、不具合の確認
⑩ 併用薬、併用療法

※NRS スコアは来院前 4 週間の子宮内膜症骨盤痛を被験者に思い出してもらうことによって評価する。

7.1.2.6. 20 週後

① 体重、BMI
② バイタルサイン（収縮期血圧/拡張期血圧・体温）
③ 自他覚症状（疼痛関連症状）
④ NRS スコア（16 週から 20 週までの平均値）※
⑤ NRS スコア（16 週から 20 週における月経期間の最大値及び平均値）
⑥ NRS スコア（16 週から 20 週における月経期間以外の最大値及び平均値）
⑦ B&B スコア、EHP-30 スコア、EQ-5D スコア
⑧ 有害事象、不具合の確認
⑨ 併用薬、併用療法

※NRS スコアは来院前 4 週間の子宮内膜症骨盤痛を被験者に思い出してもらうことによって評価する。

7.1.2.7. 中止時

① 体重、BMI
② バイタルサイン（収縮期血圧/拡張期血圧・体温）
③ 自他覚症状（疼痛関連症状）
④ NRS スコア（中止直前の来院から中止時までの平均値）※
⑤ NRS スコア（中止直前の来院から中止時までの月経期間の最大値及び平均値）
⑥ NRS スコア（中止直前の来院から中止時までの月経期間以外の最大値及び平均値）
⑦ B&B スコア、EHP-30 スコア、EQ-5D スコア
⑧ 超音波検査（卵巣チョコレート嚢胞の有無、病変の部位、卵巣チョコレート嚢胞の個数、卵巣病変の場合はそれぞれの嚢胞の最大の直径とそれに直交する直径、直径から算出される体積（cm^3））
⑨ 有害事象、不具合の確認
⑩ 併用薬、併用療法
⑪ 中止理由

※NRS スコアは来院前 4 週間の子宮内膜症骨盤痛を被験者に思い出してもらうことによって評価する。

7.2. 患者日誌

患者日誌で収集する項目については、株式会社 Smart119 の Smart:DR という名称の患者報告アウトカムの電子データ（Electronic Patient Reported Outcome, 以下「ePRO」）システム及び紙媒体の患者日誌を用いて収集する。

研究責任医師又は研究分担医師は、Day1 までに被験者に対し Smart:DR の使用方法を伝達する。被験者は Day1 から試験終了又は中止時まで、毎日スマートホンを用い、指定された URL にアクセスし、以下の項目について入力を行う。

・記録対象日（自動入力）
・月経の有無
・機器使用回数

・本機器パッド使用枚数
・NRS スコア（入力前 24 時間の最大の痛み）
・鎮痛剤使用有無
・不具合/有害事象の有無

また、同意取得後、Day1 前日までの期間は紙媒体の患者日誌を使用し、NRS スコアのベースラインとして Day1（治療開始）前最低 28 日間のデータを取得する。同意取得後、Day1 前日までに記録された NRS スコアを、Day1 以降 ePRO において取得する NRS スコアのベースラインとして取り扱う。
なお、ePRO が使用不可となった場合等の対応については別途定める手順書に従う。

7.3. 評価の方法

7.3.1. NRS スコア

直線を〈痛みがない : 0〉から〈最悪な痛み : 10〉までの 11 段階に区切って、患者自身に現在の痛みに相応する数値を示してもらい、痛みを評価する。

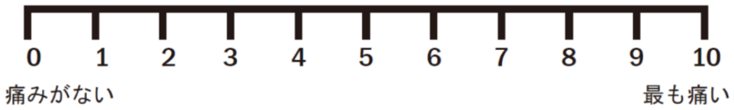

7.3.2. B&B スケール

Biberoglu and Behrman スケールは、患者が訴える 3 つの症状（月経困難症、骨盤痛、性交疼痛症）と骨盤内検査で評価する 3 つの徴候（ダグラス窩の硬結、子宮可動性の制限、骨盤の圧痛）から構成されている。それぞれ、重症度による定性評価及び、0 から 3 までの尺度で評価され、数値が高いほど症状が重いことを示している。

カテゴリー	重症度	説明
月経困難症	重度	痛みのために 1 日以上寝込み、何もできない
	中等度	1 日のうちで横になることがあり、仕事や家事はするが効率が低下
	軽度	仕事や家事の効率がやや低下
	症状なし	生理中の痛みなし
	該当せず	該当せず。過去 4 週間に生理なし
骨盤痛	重度	強力な鎮痛薬が必要。月経期間以外も、月経周期を通じて持続した
	中等度	月経周期の大部分で顕著な不快感
	軽度	ときに軽度な不快感
	なし	過去 4 週間に骨盤痛なし
性交疼痛症	重度	痛みのために性交を避ける
	中等度	性交を中断するほどの痛み
	軽度	我慢できる痛み
	症状なし	性交中に痛みなし
	該当せず	該当せず。その他の理由で性交なし

ダグラス窩の硬結の判定基準

重症度	状態
なし：0	所見なし
軽度：1	小指頭大の硬結を認める
中等度：2	「軽度」と「高度」の中間
高度：3	ダグラス窩が完全に閉塞し、弾力性が全くない

子宮可動性の制限の判定基準

重症度	状態
なし：0	所見なし
軽度：1	可動性の制限を認める
中等度：2	可動性がかなり制限される
高度：3	可動性が強く制限され、全く動かない

骨盤の圧痛の判定基準

重症度	状態
なし：0	所見なし
軽度：1	骨盤の圧痛を認める
中等度：2	「軽度」と「高度」の中間
高度：3	骨盤の圧痛を強く認める

7.3.3. EHP-30 スコア

EHP スコアは、子宮内膜症における健康関連の QOL を評価するために、患者の視点から設計された唯一の疾患別 PRO である。EHP スコアは、他の疾患別や一般的な質問票では対応できない子宮内膜症の女性の関心事を評価するための信頼性と有効性の高い調査票であると、発表されたエビデンスにより結論付けられている。

EHP は、以下の 5 つの尺度から構成されている。（括弧内の数字は、コア EHP-30（長形式）の各尺度の項目数）各項目のスコアで経時的な変化を評価する。

・痛み (11)
・コントロールと無力感 (6)
・社会的支援 (4)
・感情的な幸福感 (6)
・自己イメージ (3)

7.3.4. EQ-5D

医療技術の経済評価において質調整生存年(Quality-Adjusted Life Year; QALY)の算出に用いるための QOL 値を提供することができる指標。換算表を用いてスコア化し経時的な評価をする。

EQ-5D日本語版

- 移動の程度
  - 私は歩き回るのに問題はない ・・・1
  - 私は歩き回るのにいくらか問題がある ・・・2
  - 私はベッド（床）に寝たきりである ・・・3

- 身の回りの管理
  - 私は身の回りの管理に問題はない ・・・1
  - 私は洗面や着替えを自分でするのにいくらか問題がある ・・・2
  - 私は洗面や着替えを自分でできない ・・・3

- ふだんの活動（例：仕事、勉強、家族・余暇活動）
  - 私はふだんの活動を行うのに問題はない ・・・1
  - 私はふだんの活動を行うのにいくらか問題がある ・・・2
  - 私はふだんの活動を行うことができない ・・・3

- 痛み／不快感
  - 私は痛みや不快感はない ・・・1
  - 私は中程度の痛みや不快感がある ・・・2
  - 私はひどい痛みや不快感がある ・・・3

- 不安／ふさぎ込み
  - 私は不安でもふさぎ込んでもいない ・・・1
  - 私は中程度に不安あるいはふさぎ込んでいる ・・・2
  - 私はひどく不安あるいはふさぎ込んでいる ・・・3

8.　安全性の評価に関する取扱い

8.1. 有害事象及び不具合の定義

「有害事象」とは、同意取得後に被験者に生じたすべての好ましくない又は意図しない疾病またはその徴候(臨床検査値の異常を含む)をいい、当該試験機器との因果関係の有無は問わない。

また、「不具合」とは機器・再生医療等製品について、破損、作動不良等広く品質、安全性、性能等に関する試験機器の具合が良くないことをいう。手技によって起こったことも、不具合に含まれる。設計、交付、保管、使用のいずれの段階によるものであるかを問わない。

8.2.　疾病等の定義

疾病等とは、臨床研究の実施に起因するものと疑われる疾病、障害若しくは死亡又は感染症に加え、臨床検査値の異常や諸症状を含む臨床研究との因果関係が否定できない有害事象を指す。なお、認定臨床研究審査委員会および厚生労働大臣へ報告の必要な疾病等は、「9.3 疾病等（重篤な有害事象）報告の手順」に従い報告を行う。

8.3.　重篤な有害事象

重篤な有害事象とは、有害事象のうち、以下に該当するものをいう。

1) 死亡
2) 死亡につながるおそれ
3) 障害
4) 障害につながるおそれ
5) 入院又は入院期間の延長
6) 上記(1)～(5)に準じて重篤
7) 後世代における先天性の疾病又は異常

8.4.　非重篤な有害事象

非重篤な有害事象とは、「重篤」と判定されたもの以外の有害事象とし、その判断は研究責任医師又は研究分担医師が行う。

8.5.　報告の対象となる有害事象及び不具合

同意取得から試験終了又は中止までに発生したすべての有害事象及び不具合を収集し、試験実施に伴う因果関係の有無を評価する。

8.6.　有害事象の回復性と試験機器との因果関係

有害事象の消失とは、有害事象がない状態、又は元の状態への回復とする。有害事象における試験機器との因果関係の判定に際しては、被験者の全身状態、合併症、併用薬・併用療法、時間的関係を勘案して判断する。なお、因果関係の判断は「関連あり」、「関連なし」の 2 つの判定区分を用いる。

8.7.　有害事象の評価に必要な情報

・　有害事象の名称
・　発現日
・　消失日
・　転帰：回復、軽快、回復したが後遺症あり、未回復、死亡、不明
・　処置：	試験治療の変更なし、休止、中止、該当せず

- その他の処置：なし、薬物治療、その他
- 試験機器との因果関係：関連あり、関連なし
- 重篤度：非重篤、重篤
- 重症度：軽度、中等度、高度
  - 軽度：症状がない、または徴候もしくは症状が見られるが治療を必要としない
  - 中等度：最小限/局所的 /非侵襲的治療など何らかの治療を要する
  - 高度：重症または医学的に重大である、または全身薬物療法又は他の治療を必要とする状態、あるいは生命を脅かす状態

8.8. 不具合の評価に必要な情報

- 不具合名
- 不具合の発生日
- 不具合による疾病等発生の有無および事象名
- 疾病等の予測可能性：既知、未知
- 施行/使用期間
- 疾病等との因果関係：関連あり、おそらく関連あり、関連あるかもしれない、関連なし、不明、該当せず
- 疾病等に対する処置：あり、なし、該当せず

9. 疾病等及び重篤な有害事象発生時

9.1. 有害事象発生時の対応

研究責任医師または研究分担医師は、有害事象を認めたときは、直ちに適切な処置を行うとともに、カルテならびに症例報告書に齟齬なく記載する。また、試験機器の使用を中止した場合や、有害事象に対する治療が必要となった場合には、被験者にその旨を伝える。

9.2. 疾病等及び重篤な有害事象発生時の対応

1) 研究責任医師又は研究分担医師は、疾病等（重篤な有害事象）が発現した場合、試験機器との因果関係を問わず適切な処置を行うとともに原因の究明につとめる。
2) 研究代表医師/研究事務局は、報告内容の緊急性、重要性、影響の程度などを判断し、必要に応じて登録の一時停止や参加施設への周知事項の緊急連絡などの対策を講ずる。

9.3. 疾病等及び重篤な有害事象報告の手順

研究責任医師は有害事象が発生し、研究責任医師が報告を必要と判断した場合（8.3 重篤な有害事象）、下記の手順に従い当該有害事象情報を取り扱う。

1) 研究責任医師から実施医療機関の管理者の報告

研究責任医師は、当該有害事象情報を可能な限り速やかに実施医療機関の管理者に報告する。報告は第 1 報（緊急報告）および第 2 報（詳細報告）とする。

2) 認定臨床研究審査委員会および厚生労働大臣への報告

臨床研究法に基づき、研究責任医師が表 1 に従い認定臨床研究審査委員会への報告の必要性を判断した場合には、研究責任医師は「医薬品の疾病等報告書」（統一書式 8）を作成し、認定臨床研究審査委員会に報告する。また、当該有害事象が予測できないものである場合、jRCT を通じて、厚生労働大臣（独立行政法人医薬品医療機器総合機構）に報告する。

3) 追加情報の入手時の対応

当該有害事象が発生した臨床研究機関の研究責任医師は、当該事象に関する追加情報が得られ

た場合には、可能な限り速やかに実施医療機関の管理者および認定臨床研究審査委員会に追加報告を行う。当該追加情報の取扱いは 2)、3）の手順に準ずる。

4) 医薬品・医療機器等安全性情報報告制度に基づく対応

市販後の薬剤については医薬品・医療機器等安全性情報報告制度（医薬品、医療機器等の品質、有効性及び安全性の確保等に関する法律（昭和 35 年法律第 145 号）第 68 条の 10 第 2 項）に基づいて対応し、必要に応じて厚生労働省に報告する。

表 1　（既承認の医療機器・再生医療等製品の場合）

予測可能性	疾病等の重篤性	報告先	報告期限
予期できない	①　死亡<br>②　死亡につながるおそれ<br>③　治療のために医療機関への入院又は入院期間の延長<br>④　障害<br>⑤　障害につながるおそれ<br>⑥　上記に準じて重篤<br>⑦　後世代における先天性の疾病又は異常	認定臨床研究審査委員会	15 日
予測できる	①　死亡<br>②　死亡につながるおそれ	認定臨床研究審査委員会	15 日
予期できる	①　治療のために医療機関への入院又は入院期間の延長<br>②　障害<br>③　障害につながるおそれ<br>④　上記に準じて重篤<br>⑤　後世代における先天性の疾病又は異常	認定臨床研究審査委員会	30 日
問わない	当該特定臨床研究に用いる医療機器又は再生医療等製品の不具合の発生であって、当該不具合によって下記に掲げる疾病等が発生するおそれのあるもの<br>①　死亡<br>②　死亡につながるおそれ<br>③　治療のために医療機関への入院又は入院期間の延長<br>④　障害<br>⑤　障害につながるおそれ<br>⑥　上記に準じて重篤<br>⑦　後世代における先天性の疾病又は異常	認定臨床研究審査委員会	30 日

9.4.　有害事象の定期報告

研究責任医師は、上記の重篤な有害事象に加え、非重篤な有害事象についても定期報告にて、報告する必要がある。定期報告は、原則として、実施計画を厚生労働大臣に提出した日から起算して、一年ごとに、当該期間満了後二月以内に、認定臨床研究審査委員会の審査を受けた後、jRCT 登録により厚生局へ報告する。

9.5. 不具合発生時の報告の手順

特定臨床研究を実施する研究責任医師は、実施計画に記載された特定臨床研究の実施について、当該特定臨床研究に用いる医療機器の不具合の発生であって、当該不具合によって次に掲げる疾病等が発生するおそれのあるものについて知ったときは、「医療機器の疾病等又は不具合報告書」（統一書式 9）を作成し、これを知った日から 30 日以内にその旨を臨床研究審査委員会及び実施医療機関の管理者に報告する。

ⅰ）死亡
ⅱ）死亡につながるおそれのある疾病等
ⅲ）治療のために医療機関への入院又は入院期間の延長が必要とされる疾病等
ⅳ）障害
ⅴ）障害につながるおそれのある疾病等
ⅵ）ⅲ）からⅴ）まで並びに死亡及び死亡につながるおそれのある疾病等に準じて重篤である疾病等
ⅶ）後世代における先天性の疾病又は異常

10. 統計学的事項

本試験の統計解析計画の概要を以下にまとめた。なお、統計解析計画の詳細は、統計解析計画書に記載する。

10.1. 解析対象集団

10.1.1. 最大の解析対象集団 (full analysis set：FAS)

本試験に登録され、ランダム化後に 1 回以上試験機器が使用され、有効性データがあるすべての被験者を最大の解析対象集団 (FAS) とする。ただし、主要な登録基準を満たしていない場合（適格基準違反)、試験治療を一回も受けていない場合、ランダム化後のデータがない場合などの被験者については除外する。

10.1.2. 研究計画書に適合した対象集団 (per protocol set：PPS)

FAS から、試験方法や併用療法など研究計画書の規定に対して、以下の重大な違反があった症例を除いた被験者とする。

選択基準違反
除外基準違反
併用禁止薬違反
併用禁止療法違反

10.1.3. 安全性解析対象集団

本試験に登録され、少なくとも 1 度は試験機器が使用された症例を解析の対象とし、実際に使用された試験治療を群とする。

10.2. 目標症例数と設定根拠

解析対象例数：50 名(実機群：25 例、シャム機群：25 例)

【設定根拠】

試験機器の同型機（AT-02）を用い、線維筋痛症に対してシャム機を対照とした RCT 試験[9]を実施した結果、シャム機によるプラセボ効果は、約 14%であると推測された。

月経痛に対するオープン試験の結果、処置前の疼痛スコアが 6 以上の患者の結果について、実機 1.73（真の効果＋プラセボ効果）の疼痛減少が見られた。線維筋痛症で認められたプラセボ効果約 14%を当てはめた結果、真の効果が 1.49 であると仮定した場合、プラセボ効果は 0.24 となり、実機の標準偏差約 1.8、プラセボ効果の標準偏差約 0.3 を考慮し、サンプルサイズを計算した。

・対応のない t 検定
・両側検定
・効果量：0.969
・α ：0.05
・1-β ：0.9

その結果、βエラーについてコンサバティブに設定した場合、1 群あたりの症例数は 24 例（合

[9] A Multicenter, Prospective, Randomized, Placebo-Controlled, Double-Blind Study of a Novel Pain Management Device, AT-02, in Patients with Fibromyalgia, Pain Medicine, Volume 21, Issue 2, February 2020, Pages 326–332, Published: 26 November 2019

計 48 例）であり、脱落を考慮し、約 50 例の症例数が妥当と考えられた。

10.3. 症例の取り扱い

原則として登録された症例については、研究責任医師（研究代表医師）および統計専門家が協議の上、症例の取り扱いを決定する。新たな問題が起こった場合の症例の取り扱いについても、研究責任医師（研究代表医師）および統計専門家が、協議の上、決定する。

10.4. データの取り扱い

データ集計・解析時におけるデータの取り扱いについては、原則として以下に示す通りとする。疑義が生じた場合は、統計専門家と研究責任医師（研究代表医師）が協議の上決定する。

欠測値に対しては、必要に応じて補完を行う。試験機器使用の中止をした症例については、中止時の 1 回前のデータをもって補完する。詳細については、統計解析計画書に記載する。

10.5. 統計解析項目および解析計画

全ての症例において試験機器の使用が終了し、データが固定された後に解析を行う。全ての有効性評価において、最大の解析対象集団 (FAS) における解析を主解析とし、参考として研究計画書に合致した解析対象集団 (PPS) における解析を行う。安全性の解析は、安全性解析対象集団における解析を実施する。

統計解析の詳細はデータ固定前に別途作成する統計解析計画書に規定する。

10.5.1. 被験者背景の解析

各解析対象集団における被験者背景データの分布及び要約統計量を群ごとに算出する。名義変数については、カテゴリの頻度及び割合を群ごとに示す。連続変数については要約統計量 (例数、平均値、標準偏差、最小値、中央値、最大値) を群ごとに算出する。群間の比較には、名義変数については、Pearson のカイ 2 乗検定、ただし期待度数が 5 未満のセルが 20% 以上の場合は Fisher の直接確率計算法、連続変数については t 検定またはマンホイットニーの（U）検定を用いる。有意水準は両側 5% とする。

10.5.2. 有効性の解析

10.5.2.1. 主たる解析

治療開始前のベースラインと比較した、二重盲検期終了時における子宮内膜症関連疼痛の NRS スコアの変化量に関して、対応のない両側 t 検定（帰無仮説：AT-04 照射群の NRS 変化量 － シャム機群の NRS 変化量 ＝0 vs 対立仮説：AT-04 照射群の NRS 変化量 ― シャム機群の NRS 変化量 ≠ 0）を行い、p 値を算出する。p 値が有意水準 0.05 を下回った場合に、帰無仮説を棄却し、対立仮説を採択する。

また、感度解析として、割付因子を固定効果とした分散分析を行う。また、ノンパラメトリックなウィルコクソンの順位和検定により両群を比較する。

10.5.2.2. 副次解析

主要評価解析と同様の解析を実施する。

10.5.3. 安全性の解析

安全性の評価項目は、試験機器の不具合及び有害事象の発生頻度・割合であり、評価項目について集計表を作成し、割合の推定には 2 項分布の正確な両側 95% 信頼区間を群ごとに算出する。

必要に応じて Fisher の直接確率計算法を用いて群間比較を行う。

10.5.4. 探索的解析

主要評価項目及び副次的評価項目に関して、ベースライン値や患者背景を考慮した層別解析等を含めた探索的な解析を実施する。また、患者日誌データについて、NRS スコアの推移等を探索的に集計解析する。中止脱落をイベントとした生命表解析も行う。

10.5.5. 中間解析

本試験において中間解析は行わない。

10.6. 独立データモニタリング委員会

本試験では独立データモニタリング委員会を設置する。独立データモニタリング委員会は自ら試験を実施する者と独立した機関として設立され、本試験とは独立した立場である 2 人以上の専門家による委員で構成される。独立データモニタリング委員会は、患者の安全性を確保することを目的に、必要に応じて、被験治療及び対照における有害事象発現率の比較、重篤な有害事象に関する詳細な検討等の安全性モニタリングを行う。ときにその結果を踏まえて有害事象のリスクを軽減する為に、組入れ基準の変更等の試験デザインの変更を勧告すること、あるいは試験の継続の可否を判断することもある。

10.7. 最終解析

治療期間終了後、データが得られ症例が固定された後に解析を行う。統計解析責任者が「解析報告書」をまとめ、研究責任医師（研究代表医師）に提出する。

11. 研究計画書の遵守および不適合

(1) 研究責任医師又は研究分担医師は、臨床研究法及び関連通知、研究計画書から逸脱した行為を不適合として理由のいかんによらずすべて記録し、速やかに実施医療機関の管理者に報告する。

(2) 不適合のうち、以下に記載するような特に重大なものが判明した場合には、速やかに認定臨床研究審査委員会の意見を聞く。ただし、被験者の緊急の危険を回避するためその他医療上やむを得ない理由により研究計画書に従わなかったものについては含まない。
- 同意を取得せず試験介入を行った
- 選択基準又は除外基準違反症例を試験に登録した
- 併用禁止薬・併用禁止療法を使用した　など

12. 研究計画書、同意説明文書又は解析計画に関する変更

12.1. 研究計画書および同意説明文書の改訂

研究計画書及び同意説明文書を改訂する場合には、研究代表医師は研究計画書改訂版及び同意説明文書改訂版を速やかに認定臨床研究審査委員会に提出し、承認後、各実施医療機関の管理者の許可を得る必要がある。

12.2. 統計解析計画の変更

統計解析責任者は、統計解析計画書の内容を変更した場合、変更内容をすべて本試験の統計解析報告書に記載する。その後、研究責任医師は統計解析計画書の改訂版を速やかに認定臨床研究審査委員会に提出し、承認後、各実施医療機関の管理者の許可を得る必要がある。なお、統計解析計画書の変更は、その経緯を記録に残す。また、臨床研究の総括報告書においても統計解析計画書の変更を説明する。

13. 試験の中止または終了

13.1. 試験全体での中止の基準

責任医師（研究代表医師）は、以下の情報が得られ、試験全体の続行が困難であると考えられる時には、研究責任医師と試験全体の中止について協議のうえ、試験全体での中止を判断する。

(1) 予期できない重篤な疾病等の発生
(2) 予期できる重篤な疾病等の発生件数、発生頻度、発生条件等の発生傾向が添付文書から予測できないことを示す情報
(3) 重篤な有害事象のうち因果関係がないと判断されていたが、その後発生数、発生頻度、発生条件等の発生傾向から因果関係が否定できないと判断される情報
(4) 疾病等の発生数、発生頻度、発生条件等の発生傾向が著しく変化したことを示す研究報告
(5) がんその他の重大な疾病、障害もしくは死亡が発生するおそれがあることを示す研究報告
(6) 当該研究で有効性が認められないことを示唆する情報
(7) 試験の対象となる疾患に対して効能もしくは効果を有していないことを示す情報
(8) 試験機器等と同一効果を有する市販品について、製造、輸入又は販売の中止、回収、廃棄その他の保健衛生上の危害の発生又は拡大を防止するための措置の実施の情報

13.2. 試験全体での中止する場合の手続き

研究代表医師は、他の研究責任医師と協議のうえ試験全体を中止する場合には、その旨を認定臨床研究審査委員会に通知するとともに、厚生労働大臣に届け出る。また、投与中の被験者に対して速やかにその旨を伝え、適切な治療への変更等の適切な処理を行うものとする。

13.3. 試験の終了

研究責任医師（研究代表医師）は、試験を終了する際には、以下のことを行う。

(1) 実施計画に掲げる主たる評価項目に係るデータの収集を行うための期間が終了したときは原則としてその日から 1 年以内に主要評価項目報告書を作成する。また、臨床研究の内容に関する事項として記載した全ての評価項目に係るデータの収集を行うための期間が終了したときは、原則としてその日から 1 年以内に研究計画書につき一の総括報告書及びその概要を、それぞれ作成する。 ただし、主要評価項目報告書及び総括報告書を作成しなければならない時期が同時期の場合は、総括報告書の作成により主要評価項目報告書の作成をしたものとみなす。
(2) 前項の規定により主要評価項目報告書の作成を行う場合は、実施計画の特定臨床研究の実施状況の確認に関する事項等の変更を行う。
(3) 主要評価項目報告書、総括報告書及びその概要について、認定臨床研究審査委員会の意見を聴くとともに、病院長に提出する。
(4) 認定臨床研究審査委員会が意見を述べた日から起算して 1 ヶ月以内に、主要評価項目報告書又は総括報告書の概要、研究計画書及び統計解析計画書を jRCT にて公表する。

14. データマネジメント

14.1. データ登録の方法及び管理方法

研究責任医師（研究代表医師）又は研究分担医師は、21 CFR Part 11、臨床研究法及び ER/ES 指針の要件に対応した Electronic Data Capture（以下、「EDC」という。）を用いた症例報告書を作成する。研究責任医師又は研究分担医師は、症例報告書の記載内容の変更、修正又は追記に当たっては、症例報告書を作成した EDC 上で行い、全て電子情報として記録する。なお、研究責任医師は、研究分担医師が症例報告書を作成した場合並びに研究協力者が原資料（原データ）から症例報告書に転記した場合には、当該症例報告書が固定される前にその内容について点検し、問題がないことを確認する。試験終了時、研究責任医師に、電子症例報告書の電子媒体（例：CD-R 等）が提供される。研究責任医師は、電子症例報告書の見読性、保存性を担保する。

EDC システムの使用にあたり、実施医療機関は EDC のトレーニングを受講し、入力方法の詳細は別途入力マニュアルを参照する。

本試験における外部データは ePRO 及び紙の患者日誌とし、収集項目及び収集方法等については別途手順を定め、それに従うこととする。

14.2. 症例報告書に直接記載され、かつ原資料(原データ)と解すべき資料の特定

本試験においては、以下の文書などを原資料(原データ)とする。

(1) 被験者の同意及び被験者への情報提供に関する記録

診療録、看護記録、臨床検査データ及び画像検査フィルム等症例報告書作成の基となった記録。なお、電子カルテに格納されたデータも原資料とみなす。

(2) 試験機器使用に関する記録

(3) 本試験に関連する臨床研究法上必要な試験に係る文書又は記録

症例報告書および ePRO に記載されたデータのうち、以下に示す項目は症例報告書の記載をもって原資料(原データ)とする。ただし、診療録等に記載のある場合は、当該診療録等を原資料(原データ)とみなす。

(1) 併用薬・併用療法の目的

(2) 有害事象の程度、転帰(追跡調査時の結果を含む)、重篤度、本試験機器との因果関係の判定及び判定根拠

(3) 被験者の試験中止理由

(4) 研究責任医師又は研究分担医師のコメント

(5) 被験者の連日の症状記録（ePRO)

15. 原資料及びその他の記録の保存

研究責任医師は、試験の実施に関連する文書（申請書類の控え、病院長からの通知文書、各種申請書・報告書の控え、被験者識別コードのリスト、同意書、症例報告書などの控え、その他データの信頼性を保証するのに必要な書類または記録など）を保存する。

研究責任医師は、当該研究に係る情報等について、少なくとも当該研究の終了後 5 年を経過した日まで、適切に保存しなければならない。

16. 試料・情報等の保存及び他機関等の試料等の利用

保管場所は、千葉大学大学院医学研究院の生殖医学とし、研究室内のパスワードが設定されているコンピューターでデータを保管する。また、紙資料のうち、同意文書などのコード化しない書類等に関しては、生殖医学の鍵のかかる部屋に保管する。研究室の入り口は施錠されているため、外部の者は入室することができない。保管期間は研究終了後少なくとも 5 年間とする。保管期間終了後の文書はシュレッダーを用いて廃棄する。

17. 原資料の直接閲覧

研究責任医師は、モニタリング、監査及び認定臨床研究審査委員会又は規制当局による調査の際に、原資料等すべての臨床研究関連記録を直接閲覧できることを保証し、試験が適切に実施されていること及びデータの信頼性が十分に確保されていることを確認する。

18. 試験の品質管理及び品質保証

18.1. 品質管理

本研究において、対象者の人権が保護され、安全に、かつ研究計画書に従って実施されているか、データが正確に集積されているかを確認する目的でモニタリングを行う。モニタリングは、モニタリングに関する標準業務手順書に基づいて行う。また、リスクに基づき、施設における品質管理（オンサイト・オフサイトモニタリング）を別途定める手順書に従って行う。モニタリングを担当する者は、疾病等、不適合等の重要な発見事項、又は事実関係等を要約した内容を含む報告書を作成し、当該モニタリングの結果を研究責任医師（研究代表医師）に報告しなければならない。また、業務上知りえた情報を正当な理由なく漏らしてはならない。その業務に従事しなくなった後も同様とする。

18.2. 品質保証

本試験では承認を受けた医療機器を用いるため、新たなリスクはないと考え、監査は実施しない。

19. 倫理

本研究は「ヘルシンキ宣言」の倫理的原則に基づき、「臨床研究法」 および関連通知ならびに実施計画および本研究計画書を遵守して実施する。

19.1. 本研究の参加に伴って予想されうる利益

本研究では、子宮内膜症に伴う骨盤痛等の疼痛軽減の効果が期待されている。慢性的な疼痛を改善するため、研究対象者にとって侵襲性がなく、安全性が高い本機器を頻繁な通院を課することなく在宅にて治療可能になることは、研究対象者の時間的負担を軽減することが期待される。

19.2. 本研究の参加に伴って予想されうる不利益

1）起こりうるリスク

先行研究では、導子を皮膚に接着するための専用粘着シートによる皮膚障害を認めたが、機器に由来する有害事象の発現は認めなかった。そのため、本試験で使用する医療機器自体による有害事象の発現の可能性は極めて低いと考えられる。

2）介入によって生じる負担

シャム機に割り当てられた場合、試験機器 AT-04 本来の効果を享受することが出来ない可能性がある。

3）リスク及び負担を最小化する方法

有害事象のリスクや不利益を最小化するために、重篤な有害事象や予期されない有害事象が生じた場合には、参加施設への周知など必要な対策が講じられる体制が取られている。試験治療に関して十分な情報提供のもと同意を取得した被験者のみで本試験は行われる。

また、シャム機に割り当てられた被験者にも 16 週の使用に引き続き、実機（AT-04）が再割付され、治療が継続される。

19.3. 総合的な評価

本試験では 19.2 に記載したリスクや負担を最小化することにより被験者の安全性を確保するとともに、シャム機に割り当てられた被験者についても 16 週以降実機を使用することが可能となり、全ての被験者が、試験参加により疼痛軽減の効果を期待できることが予想されるため、利益が不利益を上回ると予想される。

20. 被験者の秘密の保全

研究責任医師は被験者の個人情報が守られていることを確認しなければならない。

(1) 症例報告書では被験者の識別は固有の被験者識別番号のみで行う。

(2) 被験者の個人情報が記載された同意文書などの書類は研究担当医師が機密文書として扱う。

21. 認定臨床研究審査委員会の審査等

認定臨床研究審査委員会は、本試験が対象者の生命、健康及び人権を尊重するものであり、下記の基本理念に基づいて実施されることを審査する。

1) 社会的及び学術的意義を有する臨床研究を実施すること
2) 臨床研究の分野の特性に応じた科学的合理性を確保すること
3) 臨床研究により得られる利益及び臨床研究の対象者への負担その他の不利益を比較考量する

こと
4) 独立した公正な立場における審査意見業務を行う認定臨床研究審査委員会の審査を受けていること
5) 臨床研究の対象者への事前の十分な説明を行うとともに、自由な意思に基づく同意を得ること
6) 社会的に特別な配慮を必要とする者について、必要かつ適切な措置を講ずること
7) 臨床研究に利用する個人情報を適正に管理すること
8) 臨床研究の質及び透明性を確保すること

22. 健康被害補償及び保険

22.1. 健康被害の補償

健康被害については、責任医師等はその回復に努め適切な医療を提供するものとする。

22.2. 賠償保険への加入

賠償責任に備え、研究責任医師および研究分担医師は賠償責任保険に加入する。

22.3. 臨床研究保険（補償保険）への加入

被験者の健康被害への補償責任に備え、研究責任医師および研究分担医師は臨床研究保険（補償保険）に加入する。

23. 金銭の支払い

試験機器および検査の費用は研究費により賄われる。試験参加に伴って特別に費用負担が増加することはなく、原疾患、他の合併症に対する検査・処置・治療は健康保険の範囲内で行われる。負担軽減費ついては、本試験に参加することにより通院頻度が増加することなどの理由から、各実施医療機関の判断により負担軽減費を支払うことを可能とする。なお、金額については 1 回の来院につき 7000 円、7 来院分を最大とし、スクリーニング、Day1、4 週、8 週、12 週、16 週、20 週などを支払いの対象とする。

24. 研究資金および利益の衝突

本試験は、令和 4 年度成長型中小企業等研究開発支援事業(Go-Tech 事業)の研究助成を得て実施する。株式会社 P・マインドと千葉大学は共同研究契約を結ぶ。P・マインドからは試験機器の提供を受ける。また、P・マインドは Go-Tech 事業により得た研究助成によって千葉大学に資金を提供する。本試験は千葉大学の研究責任医師と研究分担医師により公正に行われる。また、研究資金については、資金計画に基づいて運用され、責任医師によって適切に管理される。本研究の利害関係については、千葉大学医学部附属病院利益相反委員会の承認を得た上で、本学附属病院「臨床研究に関する利益相反ポリシー」に従い、適切に利益相反のマネジメントを行い、また、当該研究(試験)の経過を定期的に利益相反委員会へ報告等行うことにより、本研究の利害関係についての公正性を保つ。なお、著作権・データなどの知見・情報等の財産権は千葉大学に帰属するが、P・マインドの技術情報を利用して生じた発明・考案については千葉大学と P・マインドの間で別途持

分・取り扱いを協議する。

25. 研究に関する情報公開

試験責任医師は、当該臨床試験について、臨床研究等提出・公開システム（japan registry of clinical trials: jRCT）（https://jrct.niph.go.jp/）の公開データベースに、 当該研究の概要をその実施に先立って登録し、実施計画の変更及び試験の進捗に応じて適宜更新する。公開する文書は研究計画書、同意説明文書、統計解析計画書、総括報告書の概要とする。また、臨床試験を終了したときは、遅滞なく、当該臨床試験の結果を登録する。

26. 結果の公表

26.1. 公表の方法

研究責任医師は、試験を終了したときは、遅滞なく、被験者等及びその関係者の人権又は研究者等及びその関係者の権利利益の保護のために必要な措置を講じた上で、当該試験の結果を公表する。結果の公表方法としては、学会発表や論文掲載、公開データベースへの登録等、jRCT における研究計画書、統計解析計画書、統括報告書等を含む。

26.2. 公表についての取り決め

主たる公表論文は英文誌に投稿する。

研究代表医師による、研究のエンドポイントの解析結果を含まない、研究の紹介目的の学会・論文（総説）発表や、登録終了後の患者背景の分布や安全性データの学会・論文発表は研究責任（代表）医師等の了承を得て行うことができる。これらに該当しない、主たる解析と最終解析以外の発表については、事前に研究代表医師の承認を得た場合を除いて行わない。原則として、研究結果の主たる公表論文（primary endpoint の結果を初めて公表する論文）の著者は筆頭または最終著者を研究代表医師とし、論文の投稿規定による制限に従って、登録数の多い順に貢献度の高かった施設研究者を施設毎に選んで共著者とする。その他の担当者を著者に含めるかどうかは、貢献度に応じて研究代表医師が決定する。主たる公表論文以外の論文（Secondary endpoints に関する論文、副次的解析の論文など）の著者は、研究代表医師が決定する。

すべての共著者は、投稿前に論文内容をレビューし、発表内容に合意した者のみとする。内容に関して、議論にても合意が得られない場合、研究代表医師はその研究者を共著者に含めないことができる。

主たる学会発表（primary endpoint の結果の初めての学会発表）の筆頭演者は原則として研究代表医師とする。その他の学会発表は複数回に及ぶ可能性があるため、研究代表医師、登録の多い施設の研究責任医師の中から、持ち回りで発表を行うこととする。

発表者は研究代表医師の了承を得て決定する。ただし、学会発表に際しては、発表準備および発表内容について研究代表医師が責任を持ち、原則として統計解析担当者との連絡は試験調整事務局が行う。研究代表医師以外の発表者が、研究代表医師と統計解析責任者、データセンターの了承なく、直接データセンターから集計・解析結果を受け取ることはできない。

27. 試験実施体制

1. 研究責任（代表）医師
千葉大学大学院医学研究院　生殖医学　教授
連絡先：〒260-8677 千葉市中央区亥鼻 1-8-1　Tel: 043-222-7171
甲賀　かをり

2. 研究代表医師及び研究責任医師以外の研究を総括する者
千葉大学大学院医学研究院　生殖医学　診療准教授
連絡先：〒260-8677 千葉市中央区亥鼻 1-8-1　Tel: 043-222-7171
石川　博士

3. 研究・開発計画支援担当者
千葉大学医学部附属病院　臨床試験部長
連絡先：〒260-8677 千葉市中央区亥鼻 1-8-1　Tel: 043-222-7171
花岡　英紀

4. 調整管理実務担当者
千葉大学医学部附属病院　臨床試験部長
連絡先：〒260-8677 千葉市中央区亥鼻 1-8-1　Tel: 043-222-7171
花岡　英紀

5. 研究実施予定機関および責任医師
1) 千葉大学医学部附属病院　婦人科　教授
連絡先：〒260-8677 千葉市中央区亥鼻 1-8-1 Tel: 043-222-7171　甲賀　かをり
予定症例数：5 例

2) 東京大学医学部附属病院　女性診療科産科　助教
連絡先：〒113-8655 東京都文京区本郷 7-3-1　泉　玄太郎
予定症例数：10 例

3) 山梨大学医学部附属病院　産婦人科　教授
連絡先：〒409-3898 山梨県中央市下河東 1110　吉野　修
予定症例数：5 例

4) 福岡大学医学部　産科婦人科学講座　主任教授　診療部長
連絡先：〒814-0180　福岡県 福岡市城南区七隈 7-45-1　四元　房典
予定症例数：5 例

5) よこすか内科小児科・はるこレディースクリニック　婦人科　副院長
連絡先：〒292-0009　木更津市金田東 6-47-21　横須賀　治子
予定症例数：10

6) 聖順会ジュノ・ヴェスタクリニック八田　院長
連絡先：〒270-2267　千葉県松戸市牧の原 2 番地 92　八田　真理子

予定症例数：5

7) 医療法人ヒューマンリプロダクション　つばきウイメンズクリニック　院長
連絡先：〒791-1104　愛媛県松山市北土居 5-11-7　鍋田　基生
予定症例数：5

8) 医療法人かしわ会　かしわざき産婦人科　院長
連絡先：〒330-0855　埼玉県さいたま市大宮区上小町 604-4　柏崎　祐士
予定症例数：5

6. 臨床研究に関連する臨床検査施設並びに技術的部門・機関
該当なし

7. 開発業務委託機関
株式会社 Smart119（患者報告アウトカムの電子データ（ePRO）の収集）
連絡先：〒260-0013 千葉県千葉市中央区中央 2 丁目 5-1 千葉中央ツインビル 2 号館 7 階
Tel: 043-312-7471

8. モニタリング責任者
千葉大学医学部附属病院　臨床試験部 モニタリング室
連絡先：〒260-8677 千葉市中央区亥鼻 1-8-1　Tel:043-222-7171
樋掛　民樹

9. 監査
該当なし（本試験では監査を実施しない）

10. 症例登録・割付責任者
千葉大学医学部附属病院 データセンター
連絡先：〒260-8677　千葉市中央区亥鼻 1-8-1
Tel:043-222-7171　受付 FAX 番号：043-226-2644　服部　洋子

11. データマネジメント
千葉大学医学部附属病院 臨床試験部 データマネジメント室
連絡先：〒260-8677 千葉市中央区亥鼻 1-8-1　Tel:043-222-7171
花輪　道子

12. 統計解析責任者
千葉大学医学部附属病院　臨床試験部　生物統計室
連絡先：〒260-8677 千葉市中央区亥鼻 1-8-1　Tel:043-222-7171
稲葉　洋介

13. プロトコル評価専門部会
【臨床試験企画専門家】
千葉大学医学部附属病院　臨床研究開発推進センター　菅原　岳史
【医学専門家】

千葉大学医学部附属病院　臨床試験部　古田　俊介

14. 独立データモニタリング委員会

国際医療福祉大学成田病院 産科婦人科　永松　健
連絡先：〒286-8520 千葉県成田市畑ケ田 852　Tel:0476-35-5600

琉球大学医学部附属病院産婦人科　銘苅　桂子
連絡先：〒903-0215 沖縄県中頭郡西原町上原 207　Tel:098-895-1177

15. 試験機器の提供

株式会社 P・マインド
連絡先：〒101-0052 千代田区神田小川町 3-28-5 alex 御茶ノ水 P202　Tel:090-2717-0496

28. 参考資料・文献リスト

1) 難治性希少部位子宮内膜症の集学的治療のための分類・診断・治療ガイドライン
2) 試験機器概要書
3) A Multicenter, Prospective, Randomized, Placebo-Controlled, Double-Blind Study of a Novel Pain Management Device, AT-02, in Patients with Fibromyalgia, Pain Medicine, Volume 21, Issue 2, February 2020, Pages 326–332, Published: 26 November 2019
4) 政府統計による子宮内膜症患者数
http://www.mhlw.go.jp/toukei/saikin/hw/kanja/10syoubyo/dl/h26syobyo.pdf
5) 公益社団法人　日本産婦人科学会　研修ノート（4）子宮内膜症への対応
6) 試験機器概要書
7) エイト　添付文書
https://www.info.pmda.go.jp/downfiles/md/PDF/650949/650949_30400BZX00015000_A_01_02.pdf
8) 試験機器概要書、エイト添付文書
9) A Multicenter, Prospective, Randomized, Placebo-Controlled, Double-Blind Study of a Novel Pain Management Device, AT-02, in Patients with Fibromyalgia, Pain Medicine, Volume 21, Issue 2, February 2020, Pages 326–332, Published: 26 November 2019

添付資料　エイト添付文書

添付文書

2022 年 3 月(新様式第 1 版)　　　承認番号:30400BZX00015000

機械器具(12) 理学診療用器具
管理医療機器　交番磁界治療器　(71097002)
特定保守管理医療機器

エイト

【禁忌・禁止】

1. 併用医療機器「相互作用の項参照」
(1) 人工心肺、ペースメーカ等の生命維持用医用電気機器と併用しないこと(磁界により当該電気機器が誤作動するおそれがある)
(2) 心電計等の装着型医用電気機器と併用しないこと(磁界により当該電気機器が誤作動するおそれがある)

【形状・構造及び原理等】

＜概要＞

本品は、導子(パッド)の内部にあるコイルから発生させた 2 種類の交番磁界(2 kHz 及び 83.3MHz)を用い、体外から疼痛部等に照射させることで疼痛を緩和させる磁気治療装置である。

＜構成＞

- 磁気治療装置　1 台
- 導子(パッド)　1 組(4 個)

＜形状、寸法等＞

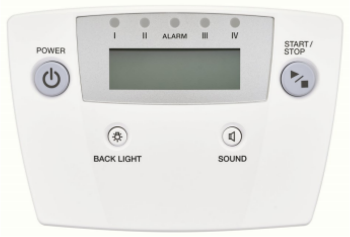

本体

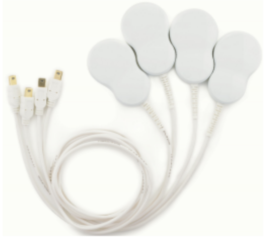

導子(パッド)

本体(縦×横×厚さ):100×148×35 mm
導子(パッド)(縦×横×厚さ):64×42×15 mm
ケーブル長さ:900 mm

＜原材料＞

ABS 樹脂

＜機器の分類＞

内部電源機器、B 形装着部

＜電気的定格＞

電源:DC 2.4～3.0 V

＜原理＞

導子(パッド)内に位置するコイルから発生させた 2 種類の交番磁界(2 kHz 及び 83.3 MHz)を疼痛部等に照射することにより疼痛を緩和させる。

【使用目的又は効果】

本品は、2 種類の交番磁界を経皮的に照射し、神経を刺激することで疼痛を緩和させるために用いられる。

【使用方法等】

1. 使用前の準備
(1) 事前の確認
1) 構成品がすべて揃っていることを確認する。
2) 取扱説明書に従い、本品の動作を確認する。

2. 使用方法
(1) 治療の開始／終了
1) 粘着シートの機器側ライナーを剥がし、導子(パッド)に貼付する。
2) 本体の電源スイッチを 1 秒以上長押しし、電源を入れる。
3) 接続ポートにパッド接続用コネクタを接続する。
4) パッドに貼付した粘着シートの皮膚側ライナーを剥がし、パッドを治療部位に貼付する。
5) スタート／ストップスイッチを 1 秒以上長押しし、治療を開始する。
6) 30 分経過すると液晶画面に治療終了の旨が表示され、自動で停止する。
7) 1 回あたり 30 分以上使用する場合は、再度 5)から開始する。
8) 電源スイッチを 1 秒以上長押しし、電源を切る。
9) 皮膚からパッドを取り外した後、パッドから粘着シートを剥がし、次の使用に備えてパッドを清潔に保つ。
(2) 治療の中止
1) 治療中に本品の使用を中止したい場合は、スタート／ストップボタンを 1 秒以上長押しする。
2) 電源スイッチを 1 秒以上長押しして電源を切る。

3. 使用方法等に関連する使用上の注意
(1) 粘着シートを使用しない場合は、パッドを疼痛部に当ててパッド自体を上から固定してもよい。
(2) 1 回あたり 30 分以上、1 日 1 回以上使用すること。ただし、1 日の合計使用時間が 2 時間を超えないようにすること。

【使用上の注意】

1. 使用注意(次の患者には慎重に適用すること)
(1) 妊娠している人、その可能性のある人又は授乳中の人[体調不良を起こす可能性がある]
(2) 適用部位の皮膚に異常のある人[粘着シートにより皮膚の異常を悪化させる可能性がある]
(3) 電磁波アレルギーのある人

取扱説明書を必ずご参照ください。

1/2

(4) その他医師の治療を受けている人や特に身体に異常を感じている人[体調不良を起こす可能性がある]

2. 重要な基本的注意

(1) 自動車等を運転しながら使用しないこと。

(2) 睡眠時に使用しないこと。

(3) 医師に運動を制限されている場合、運動中に使用しないこと。

(4) スポーツや登山など、ウォーキングを超える激しい運動中に使用しないこと。

(5) 酒気を帯びた状態で使用しないこと。

(6) 湿度の高いところや入浴しながら使用しないこと。

(7) 高齢者、身体の不自由な方は、保護者や付添いなしで使用しないこと。

(8) 皮膚に異常を感じた場合は、使用を中止し医師に相談すること。

(9) パッドを患部にあてたまま放置しないこと。

(10) 次のような場所で使用しないこと。

1) 使用環境温湿度以外の場所(直射日光のあたる場所、車内など)

2) 強電磁界下(変電施設や携帯電話の基地局の近く、電気毛布、電気カーペット、磁器ベルトや磁界を発生させる他の医療機器の近くで使用する場合など)

3) ほこりの多い場所

4) 火気の近く

5) 過度な振動や落下の可能性がある不安定な場所

6) 化学薬品の保管場所や、腐食性ガスの発生する場所

3. 相互作用

[併用禁忌](併用しないこと)

医療機器の名称等	臨床症状・措置方法	機序・危険因子
人工心肺、ペースメーカ等の生命維持用医用電気機器	使用禁止	磁界により生命維持用電気機器が誤作動するおそれがある
心電計等の装着型医用電気機器	使用禁止	磁界により装着型医用電気機器が誤作動するおそれがある

4. 不具合・有害事象

本品の使用により、以下の不具合又は有害事象が生じる可能性がある。

＜不具合＞

- 機器の破損・故障
- 導子の破損・故障
- 電池カバーの破損
- 発熱
- パッドの落下
- パッドの変形
- パッドへの粘着剤の付着
- 効果の低下
- 動作停止

＜有害事象＞

- 熱傷
- 皮膚の炎症・皮膚疾患
- 熱感・発汗・発赤(局所)
- 掻痒感
- 痛みの増悪
- 頭痛

5. 妊婦、産婦、授乳婦及び小児等への適用

妊婦、産婦、授乳婦及び小児については使用経験がなく、安全性が確立されていない。

【臨床成績】

本品の有効性及び安全性を評価するため、新規に急性腰痛を有すると診断された患者、あるいは以前に同疾患と診断され既に何らかの治療を受けている患者を対象として、前向き、多施設共同、単群非盲検試験が実施された。国内2施設において、30症例の被験者が登録された。

1. 有効性評価

主要有効性評価項目である本治験登録時のVAS値と治療期間(4週間)終了日のVAS値の差は、FAS(最大解析対象集団:n=30)で36.95±22.91 mm、PPS(治験実施計画書に適合した解析対象集団:n=29)で37.09±23.30 mmであった。

2. 安全性評価

本治験では、機器の不具合の有無と発生頻度、重篤な有害事象を含む有害事象の有無と発現頻度について評価した。その結果、機器の不具合、並びに重篤な有害事象は認められなかった。有害事象は、安全性解析対象集団(30例)において9例9件(30.0 %)に発現した。全9例中、機器との因果関係が否定できない有害事象は、一般・全身障害及び投与部位の状態(医療機器使用部位反応)が4例4件(13.3 %)であった(表1)。当該有害事象は専用粘着シートによるかぶれ・掻痒感であった。

表1. 有害事象

有害事象名	発生頻度(発生件数)
一般・全身障害及び投与部位の状態<br>(医療機器使用部位反応)	13.3 % (4/30)

【保管方法及び有効期間等】

1. 保管条件

周囲温度:−20～60℃

相対湿度:30～96%(結露なきこと)

2 .動作保証温度

周囲温度:0～35℃

3. 耐用期間

5年[自己認証(当社データ)による]

(ただし、指定された使用環境において標準的な頻度で使用され、指定の保守点検と定期交換部品・消耗品の交換をした場合の年数であり、使用状況によっては異なる場合がある)

【保守・点検に係る事項】

＜使用者による保守点検事項＞

1. 目視による点検

(1) 外観の確認

機器の外観に異常がないことを確認すること。

(2) 清浄性の確認

1) 機器が清浄な状態であることを確認すること。

2) 機器の汚れは、柔らかい布でから拭きすること。

3) 汚れがひどい場合は、水又は薄めた中性洗剤をしみ込ませた布をかたく絞り、数回ふき取った後に、柔らかい布でから拭きすること。

2. 機能の確認

(1) 機器の動作確認

機器が正常な状態かつ正常な動作であることを確認すること。

なお、点検方法については取扱説明書を参照のこと。

＜業者による保守点検事項＞

2年～5年ごとの定期点検を弊社又は弊社の指定する業者に依頼すること。詳細は取扱説明書を参照すること。

【製造販売業者及び製造業者の氏名又は名称等】

製造販売業者: 株式会社P・マインド

取扱説明書を必ずご参照ください。
