# Supplementary material for: Efficacy and safety of a novel pain management device, AT-04, for endometriosis-related pain: study protocol for a phase III randomized controlled trial
Source: Reprod Health. 2024 Jan 26;21:12. doi: 10.1186/s12978-024-01739-8 (PMC10811886; doi:10.1186/s12978-024-01739-8)
Supplement: Supplementary file 2 — Additional file 2. The participant consent form has been approved by the Clinical Study Review Board of Chiba University Hospital, Chiba, Japan, and registered by the Japan Registry of Clinical Trials (jRCTs032230278, https://jrct.niph.go.jp/). An overview of this research is publicly available through the Japan Registry of Clinical Trials. [file 12978_2024_1739_MOESM2_ESM.pdf]

患者さんへ

# 臨床研究：「子宮内膜症に対する AT-04 の有効性および安全性に関するシャム機対照二重盲検並行群間比較試験」についてのご説明

研究代表医師：甲賀　かをり

千葉大学医学部附属病院　婦人科

# 内容

1．臨床研究とは？ 3
2．この臨床研究について 3
2-1．この臨床研究の目的について 3
2-2．従来の治療と試験治療について 3
3．臨床研究の方法と期間 5
３-１．あなたが対象となる理由について 5
３-２．この臨床研究の方法について 7
３-３．この臨床研究の期間と参加予定人数について 10
４．この臨床研究の実施により予期される利益と不利益について 11
５．この研究に参加しない場合の他の治療方法と予期される利益と不利益について 12
６．あなたの健康に被害が生じた場合について 12
７．この臨床研究への参加は患者さんの自由な意思によるものです。 13
８．この臨床研究に関する情報は、随時ご連絡します 13
９．この臨床研究を中止させていただく場合があります 13
10．この臨床研究への参加に同意された場合は、次の点を守って下さい 14
11．あなたの個人情報の取り扱いについて 15
11-1．あなたから得たデータがあなたのものだと特定されることはありません。 15
11-2．研究中あるいは研究終了後に、カルテなどからあなたの情報が調査されることがありますが、あなたに関する情報の秘密は守られます。 15
11-3．この研究結果が公表される場合でも、あなたの身元が明らかになることはありません 15
12．この臨床研究に関する情報公開の方法 16
13．この臨床研究の実施に関する資料の開示、閲覧について 16
14．データ等の保管および廃棄の方法と二次利用について 16
14-1．データ等の保管および廃棄の方法 16
14-2．　データ等の二次利用について 16
15．知的財産権と利益相反について 17
16．あなたの費用負担について、この臨床研究に参加中の負担を軽減するための費用について 18
17．臨床研究の審査について（臨床研究審査委員会について） 18
18．研究組織について 19
19．研究担当医師の連絡先および相談窓口（苦情および問い合わせ） 20

## 1．臨床研究とは？

臨床研究とは、人を対象に病気の原因解明や、予防・診断・治療法の改善、患者さんの生活の質の向上などを目的として行われる研究です。臨床研究により新しい治療法を確立することは医療機関の役割であり、患者さんのご協力により成し遂げることができるものです。

今回説明する臨床研究は臨床研究法に基づいて実施するもので、実際の診療に携わる医師が医学的必要性・重要性に鑑みて、立案・計画して行うものです。製薬会社などが行う新薬の安全性・有用性を調べ、厚生労働省の承認を得るための臨床試験、いわゆる治験ではありません。

この研究については、国立大学法人千葉大学臨床研究審査委員会の審議に基づき、医療機関の長の許可を得ています。また、臨床研究法第５条第１項に則り、厚生労働大臣に規定の実施計画の提出を行っています。研究に参加されるかどうかはあなたの自由意思で決めて下さい。参加されなくてもあなたが診療などで不利益を被ることはありません。

## 2．この臨床研究について

### 2-1．この臨床研究の目的について

この研究の主な目的は、子宮内膜症の患者さんに対して、磁力を利用して痛みを和らげることを目的に開発された医療機器（商品名「エイト」、試験機器番号「AT-04」）が、子宮内膜症の患者さんの痛みにも効果があるかどうかを調べることです。

### 2-2．従来の治療と試験治療について

1）あなたの病気について

あなたは、これまでの診療で、子宮内膜症という病気にかかっていることがわかっています。この病気は、子宮内膜またはそれに似た組織が、何らかの原因で、子宮の外で発生し発育してしまう病気です。

子宮内膜症の主な症状は、月経（生理）の時にあらわれる、下腹部や腰などの痛みで、この病気の患者さんの約９割が経験します。年齢とともに悪くなることが特徴です。月経の時以外に、腹痛を訴える患者さんも多くいます。排便痛や性交痛を経験する患者さんもいます。これらの痛みによる QOL（生活の質）の低下も大きな問題となっています。

2）あなたの病気に対する治療について

子宮内膜症に対する治療は、大きく分けて薬と手術があります。年齢、病気の場所や大きさ、症状、現在もしくは将来子供を持ちたいか、などによって決めます。

薬の治療は、まず、鎮痛剤（痛み止め）か漢方薬を使います。これで効果が不十分で、かつ、今すぐ子供を持つ希望がない場合は、低用量エストロゲン・プロゲステロン配合薬やプロゲスチン製剤といったホルモン剤を使います。それでも症状が続く場合は、ゴナドトロピン放出ホルモン拮抗剤（ゴナドトロピン放出ホルモンというホルモンの働きを打ち消す薬）を用います。ただし、これらの薬は効果が十分でないこと、あるいは副作用が現れることが多く、さらに治療中は妊娠ができないことから、長期の治療が難しいことが少なくありません。

このように薬で効果がない、あるいは副作用が強い場合、あるいは妊娠を希望する場合には、手術が選択されます。手術によって病気の部分だけ、あるいは病気になった臓器（卵巣など）ごと摘出します。しかし、手術を行っても痛みが改善しないことが多いのが現状です。

3）研究で使用する医療機器についての説明

本研究で用いる試験機器（AT-04）は、磁力を利用して痛みを和らげることを目的に開発されたものです。人体にほぼ無害な 2 種類の磁界（磁界の強さは地球の磁気の約 1/3 程度）を同時発生させ、それを体に当てて使う医療機器です。機器につながっているパッドを体の痛い部分にあて、痛みの緩和をはかります。これは 2022 年 1 月に疼痛緩和を目的とした医療機器として、厚生労働省より製造販売承認を取得しました。

本機器が痛みを緩和させるしくみは、動物実験などで明らかになっています。例えば、本機器をマウスのモデルに用いると、神経や脳において痛みを伝える物質を抑える効果があります。そのしくみから考えると、本機器は子宮内膜症の病気の部分やそれから生じる痛みにも効果が期待できます。さらに、本機器は、線維筋痛症（身体の広い範囲に痛みがあらわれ、身体のこわばり、激しい疲労感、不眠、頭痛やうつ気分などがおきる病気）、腰痛症などの患者さんに対する臨床試験で、すでに有効かつ安全に用いられることが明らかになっています。月経痛の患者さんに対する予備的研究でも、参加された患者さんから症状の緩和や子宮内膜症の病状の改善が報告されています。今回の臨床研究で、子宮内膜症による痛みや、子宮内膜症の病気そのものへの治療効果が確認できれば、あなたと同様の子宮内膜症の患者さんに対して有益な情報となります。

この臨床研究では、AT-04（本当の器械）の他に、「本当の器械と見た目は区別がつかないが磁界を発生しない器械」も使用します。本当の器械を「実機」と呼ぶのに対し、このような器械は「シャム機」と呼ばれます。この研究では、実機（AT-04）の効果と安全性を正確に評価するために、実機を使用するグループとシャム機を使用するグループに分かれていただきます。詳しい流れは７ページの、「3-２．この臨床研究の方法について　１）研究の流れ」で説明します。

## 3. 臨床研究の方法と期間

### ３-１．あなたが対象となる理由について

１）研究に参加いただける方

以下の条件をすべて満たす方に参加いただけます。

1. 同意取得時に、18 歳以上である方
2. 閉経していない方
3. 子宮内膜症の診断を受けている方（以下のいずれかに該当する場合。なお、術後再発した場合は、再度、以下のいずれかで診断されていること。）

①5 年以内に実施した開腹または腹腔鏡手術により子宮内膜症と診断された方

②1 年以内に実施した MRI または超音波検査（経腟、経腹または経直腸）により子宮内膜症と診断された方

③治療開始前に実施した内診・直腸診により子宮内膜症と診断された方

4. 治療開始前に子宮内膜症に由来する痛みが、中等度以上であると研究責任（分担）医師が判断した方
5. 同意取得前 28 日間の子宮内膜症による平均の痛み NRS（Numeric Rating Scale）が 4 以上である方
6. 同意取得前の２８日間において、子宮内膜症に対する治療をあらたに開始していない、または、子宮内膜症に対してこれまでに受けてきた治療内容（薬の処方内容・用法・用量を含む）を変更していない方
7. 同意取得前の 28 日間において、子宮内膜症骨盤痛の急激な悪化がないと研究責任（分担）医師が判断した方
8. 本人の自由意思により、研究の参加について文書による同意を得られている方

２）研究に参加できない方

以下に示す条件のうち、1 つでも当てはまる方は参加出来ません。

1. 同意取得８週以内に以下のような試験機器の効果に影響を及ぼす可能性がある薬剤を服用された方
   - 他の臨床試験薬、治験薬
   - GnRH アナログ製剤、ダナゾール、アロマターゼ阻害剤、選択的エストロゲン受容体モジュレーター製剤
2. 本研究で用いる試験機器（AT-04）を含む磁気を用いた治療機器を過去に使用したことがある方
3. 非ステロイド性抗炎症薬と呼ばれる痛み止めの薬剤を常用している方
4. 卵巣チョコレート嚢胞が 10cm 以上、かつ画像検査実施時の年齢が 40 歳以上の方
5. 両卵巣摘出術の既往がある方
6. 著しい不正子宮出血または原因不明の不正出血がある方
7. 試験期間中に子宮筋腫の治療が必要な方
8. 過敏性腸症候群および・または重度の間質性膀胱炎による下腹部痛がある方

9. 重度の肝障害、黄疸、腎障害、心血管系疾患、内分泌系疾患、代謝疾患、肺疾患、胃腸疾患、神経疾患、泌尿器疾患、免疫疾患、並びに精神疾患（特にうつ様症状）およびそれに起因する自殺企図等の既往がある方
10.人工心肺およびペースメーカ等の生命維持用医用電気機器を使用している方
11.心電計等の装着型医用電気機器を使用している方
12.他の医薬品や医療機器の治験や臨床試験に参加している方
13.治療のために入院の必要がある方

3）妊娠について

使用中に妊娠がわかった場合も、患者さんのご希望があれば継続していただけます。

## 3-2．この臨床研究の方法について

1）　研究の流れ

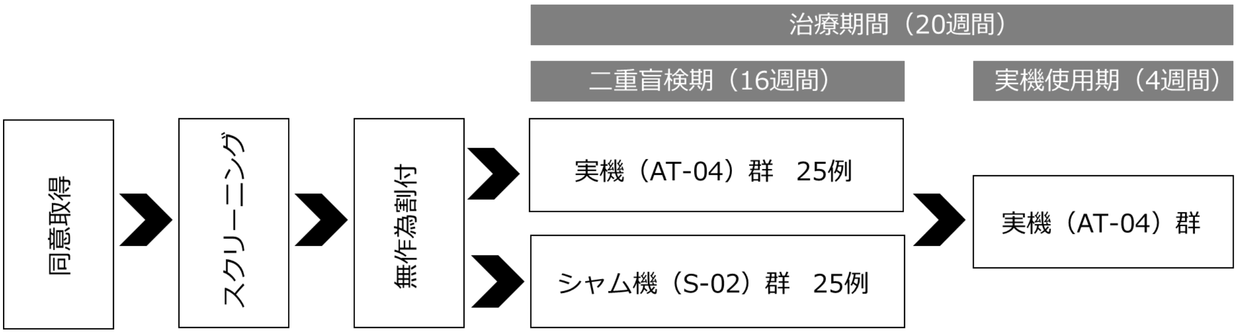


図１　研究の流れ

この研究は、二重盲検期（16 週間）、実機使用期（4 週間）の２つの期間、合わせて２０週間です。同意を取得した後、スクリーニング検査にて、研究参加の基準を満たしているか確認し、患者さんを無作為に「実機群（本当の器械を使うグループ）」、「シャム機群（本当の器械と見た目は区別つかないが磁界を発生しない器械を使うグループ）」の２つのグループに分けます（無作為とは、コンピュータによって、１：１の確率で自動的に割り振ることで、どちらになったかはあなたにも担当医師にもわかりません）。

「実機群」に割り振られた患者さんは、AT-０４（本物の機器）を 16 週間使用します。「シャム機群」に割り振られた患者さんは、S-02（シャム機）を 16 週間施術します。その後、全ての患者さんに対して AT-04 を４週間施術します。

２）研究で使用する医療機器の使用方法について

機器は、自宅で使用します。１回当たり 30 分、１日２回（60 分）以上、１日４回まで（最大２時間)、下腹部２箇所に使用します。なお、下記の図に示した基本貼付部位以外に痛みを感じる場合は、痛みを感じる部位に追加で２箇所に使用出来ます。パッドは、専用の両面テープ、市販のサージカルテープで体に固定するか、サポーター等を用い固定します。使用中は特に何も感じません。また姿勢の制限はなく、パッドがはずれなければ動いても構いません。

この研究では、16 週の来院時に機器を交換します。16 週までに使用していた機器と交換後の機器では熱さの感じ方が異なったり、多少の使用感の違いを感じることがあります。

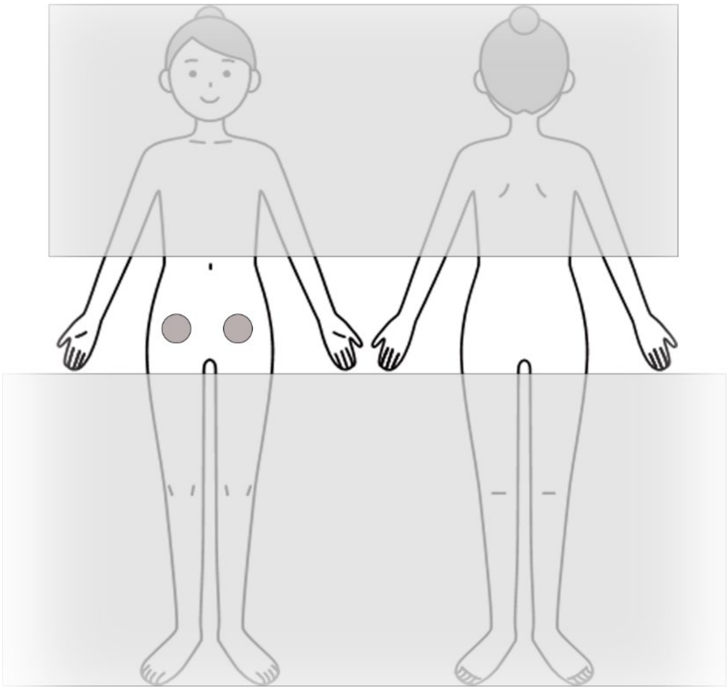

基本貼付部位（●）

この研究中は、子宮内膜症に伴う症状に対して現在投与中の薬があった場合は、そのまま継続して使用することとします。原則として、研究期間中は、薬剤の変更、用法・用量の変更は行いません。

3）スケジュール

この研究のスケジュールを表 1 に記載します。研究担当医師は、スケジュールに従って観察・検査等を実施します。また、患者さんには Day1 以降、この研究の終了又は中止まで毎日、携帯端末アプリケーションを利用した患者日誌に、その日の①月経の有無、②本機器の使用回数、③機器を使用する際に使用したパッドの使用枚数、④痛みの大きさ、⑤痛み止めの使用の有無、⑥機器の不具合又は有害事象の有無などを入力していただきます。なお、スクリーニング期間中（Day1 まで）の最大 35 日は、前述の 6 つの項目を紙の患者日誌に記録していただきます。また、以下の薬は、研究中は服用いただけません。

GnRH アナログ製剤、ダナゾール、アロマターゼ阻害剤、選択的エストロゲン受容体作動薬、麻薬性及び非麻薬性鎮痛薬（トラマドール等）、疼痛緩和目的で使用される局所麻酔薬

表１　スケジュール表

| | | スクリーニング | 二重盲検期 | | | | | 実機使用期 | 中止時 |
|---|---|---|---|---|---|---|---|---|---|
| | | | Day1 ※1 | 4 週後 | 8 週後 | 12 週後 | 16 週後 | 20 週後 | |
| 許容範囲 | | -35 | - | ±7 | ±7 | ±7 | ±7 | ±7 | - |
| 同意取得 | | ● | | | | | | | |
| 被験者背景 | 年齢 | ● | | | | | | | |
| | 身長 | ● | | | | | | | |
| | 体重・BMI | ● | | | | | ● | ● | ● |
| | バイタルサイン（血圧・体温） | ● | | ● | ● | ● | ● | ● | ● |
| | 既往歴・合併症 | ● | | | | | | | |
| | 前治療 | ● | | | | | | | |
| | 身体所見 | ● | | | | | | | |
| | 自他覚症状 | ● | | ● | ● | ● | ● | ● | ● |
| | 超音波検査 | ● | | | | | ● | | ● |
| 評価項目（疼痛） | NRS スコア ※2 | ● | ○ | ● | ● | ● | ● | ● | ● |
| | NRS スコア（月経期間） | ● | | ●※3 | ●※3 | ●※3 | ●※3 | ●※3 | ●※3 |
| | NRS スコア(月経期間以外) | ● | | ●※3 | ●※3 | ●※3 | ●※3 | ●※3 | ●※3 |
| 副次評価（子宮内膜症） | B&B スコア | ● | ○ | ● | ● | ● | ● | ● | ● |
| | EHP-30 スコア | | ○ | ● | ● | ● | ● | ● | ● |
| | EQ-5D スコア | | ○ | ● | ● | ● | ● | ● | ● |
| 有害事象 | | ― | ― | ― | ― | ― | ― | → | ● |
| 不具合 | | | ― | ― | ― | ― | ― | → | ● |
| 併用薬・併用療法 | | ― | ― | ― | ― | ― | ― | → | ● |
| 試験機器 | 登録・割付 | ● | | | | | | | |
| | 交換 | | | | | | ● | | |
| 患者日誌 | 月経の有無 | ― | ― | ― | ― | ― | ― | → | ● |
| | 機器使用回数 | ― | ― | ― | ― | ― | ― | → | ● |
| | パッド使用数 | ― | ― | ― | ― | ― | ― | → | ● |
| | NRS スコア | ― | ― | ― | ― | ― | ― | → | ● |
| | 鎮痛剤使用有無 | ― | ― | ― | ― | ― | ― | → | ● |
| | 不具合・有害事象の有無 | ― | ― | ― | ― | ― | ― | → | ● |
| 中止理由 | | | | | | | | | ● |

※1　Day1 は規定された日付もしくはその前 14 日以内に来院いただき、○で示した検査を実施します。機器による治療は Day1 より開始します。

※2　来院ごとに、来院前 4 週間の痛みを思い出し、痛みの程度を評価します。

※3　患者日誌の情報を用いて痛みの程度を評価します。

## 3-3．この臨床研究の期間と参加予定人数について

この研究の予定期間は、機器使用開始から 20 週間です。機器使用開始から二重盲検期として 16 週間、その後実機使用期として 4 週間、合計 20 週間の治療期間を予定しています。

参加予定人数は 50 名で、AT-０４群（実機群）に 25 名、S-02（シャム機群）に 25 名です。

## ４．この臨床研究の実施により予期される利益と不利益について

＜予想される利益＞

この研究で使用する治療機器は、急性腰痛の患者さんを対象とした臨床試験で安全性および有効性が確認され、疼痛を緩和させる治療機器としてすでに承認されています。この研究に参加することによる利益として、あなたの子宮内膜症およびそれにともなう痛みを緩和することが期待されます。また、この研究の結果が新たな情報を医学分野・科学分野に提供することで、将来的に同じ病気で苦しむ患者さんの役に立つかもしれません。

＜起こるかもしれない不利益＞

１）起こりうるリスク

○副作用

添付文書にも記載されている通り、以下の有害事象が発生する可能性があります。

| 予想される有害事象 |
|---|
| 熱傷、皮膚の炎症・皮膚疾患、<br>熱感・発汗・発赤（局所）、<br>掻痒感<br>痛みの増悪<br>頭痛 |

この機器を使用することにより、これまで報告されていないような症状が発生する可能性があります。

本研究で用いる磁気治療機器を用いた研究では、これまでに重大な合併症の報告はありません。しかしながら、治療開始前に完全に予測することはできません。もし合併症等が出たときや、体調がいつもと違うと感じられた場合には、適切な治療を行いますので担当医師にご連絡ください。

○その他参加に伴うリスク

この研究に参加された場合、一般診療の治療に比べて来院回数や病院の滞在時間などが増える可能性があります。また、自宅で１回 30 分、１日２回から４回（最大２時間）の治療を継続することで、パッドがずれたり、はずれたりするような激しい動作ができなくなる可能性があります。

２）生じる不便

シャム機群に割り当てられた場合、試験機器 AT-04 に期待されているような効果は享受できない可能性があります。

## ５．この研究に参加しない場合の他の治療方法と予期される利益と不利益について

この研究に参加しない場合においても、子宮内膜症の症状に対する最適な治療を継続して行います。

子宮内膜症に対する治療は、大きく分けて薬と手術があります。薬は、まず、鎮痛剤（痛み止め）か漢方薬を使います。これで効果が不十分で、かつ、今すぐ妊娠する希望がない場合は、ホルモン剤を使います。薬で効果がない、あるいは副作用が強い場合、あるいは妊娠を希望する場合には、手術が選択されます。しかし、特に痛みについては、手術を行っても改善しないことが多いのが現状です。

本研究で使用する機器は、体に対する負担が軽く、これまでの研究結果から、重大な有害事象の発現の可能性は低いと考えられます。しかし、子宮内膜症に対する効果と安全性については大規模な研究での検討はされていないため、本研究で確かめる必要があります。

## ６．あなたの健康に被害が生じた場合について

この臨床研究は、これまでの報告に基づいて科学的に計画され、慎重に行われます。もし研究の期間中あるいは終了後にあなたに副作用などの健康被害が生じた場合には、当病院において通常の保険診療の範囲内で、責任をもって医師が適切な診察と治療を行います。特別に患者さんの費用負担が増加することはありません。

健康被害が生じた場合の連絡窓口は、19．研究担当医師の連絡先および問い合わせ窓口（苦情および問い合わせ）をご参照下さい。

健康被害が生じた場合の補償責任に備え、研究担当医師は臨床研究保険（補償保険）に加入しています。

## ７．この臨床研究への参加は患者さんの自由な意思によるものです。

この研究に参加するかどうかは、あなたの自由意思によって決めてください。研究への参加を辞退することもできますし、一度参加に同意していただいた後でも、いつでもそれを撤回することができます。参加しない場合や同意を取り消した場合でも、患者さんに最も適した治療を行い、治療上の不利な扱いを受けたり、研究に参加する前に受けるべきであった利益を失ったりすることはありません。

あなたが同意を撤回した場合でも、それまでに集められたデータは研究に利用させていただきます。もし、データの利用も含めて撤回したい場合は、担当医にご相談ください。なお、撤回のタイミングですでに情報が解析されていたり、結果が発表されている場合は、あなたのデータを取り除くことはできませんのでご了承ください。

## ８．この臨床研究に関する情報は、随時ご連絡します

本臨床研究に参加されている期間中、あなたの研究参加の継続の意思に影響を与えるような情報を新たに入手した場合は、直ちにお知らせします。また、この治療法に関して重要な情報が得られた場合は、研究参加の継続に関してもう一度あなたの意思を確認します。

## ９．この臨床研究を中止させていただく場合があります

参加の同意をいただいた後でも、次のような場合には参加をお断りしたり、治療を中止したりすることがあります。治療を中止した後も、担当医師が必要であると判断した場合には、検査を受けていただく場合があります。

１）　試験機器の１日あたりの使用回数を制限しても有害事象が発現し、かつ担当医師が中止を必要と認めた場合

２）　試験機器の使用継続が困難な有害事象が発現し、かつ担当医師が中止を必要と認めた場合

３）　あなた（もしくは代理の方）が研究への参加中止を申し出た場合

４）　その他、担当医師があなたの試験継続が不可能と判断した場合

５）　研究代表医師または研究責任医師が中止の決定を判断した場合

６）　この研究全体が中止となった場合

## 10. この臨床研究への参加に同意された場合は、次の点を守って下さい

１）以下の事項は、研究参加中の安全のため、また正確な研究データを集めるために必要なことですのでお守りください。

① 使用方法を守って使用してください

② 担当医師から指示された来院日は特別な事情がない限り守ってください

③ 適切な方法で避妊してください

④ 他の病院や診療科で診察を受ける場合や受けた場合は連絡してください

⑤ 臨床研究中に薬を使用する場合は、前もってご相談ください。現在使用している薬（市販薬・健康食品を含む）がある場合や、研究参加後に新しく薬を使用する場合には、治療の効果判定に影響を及ぼす可能性もあるため、前もって担当医師にご相談ください。

⑥ いつもと体調が違うと感じられた場合は、いつでも担当医師までご連絡ください。

⑦ 住所や電話番号など連絡先が変更になる場合は、必ず担当医師までお知らせください。

⑧ 研究の参加をやめる場合、中止時の検査のため来院してください。あなたご自身の安全性を確認するためにも大変重要となります。

２）他科・他院に通院している薬局でお薬を購入している場合には、下記事項をお守りください。

　現在、あなたが他の病院に通院されている場合は、その病院と病名、使用しているお薬をお知らせください。また、薬局等で購入して使用しているお薬がある場合もお知らせください。これらは、研究を安全に行うために大切なことです。

　また、あなたが他の病院に通院されている場合は、この研究に参加していることをその病院にお知らせすることがあります。他院におけるあなたの診療情報を

ご提供いただくことがありますので、ご了承ください。その際にはあらためてご連絡します。

## 11．あなたの個人情報の取り扱いについて

あなたが同意書に署名されますと、あなたの情報を収集、閲覧および利用することにご承諾いただけたことになります。その場合にも、あなたの名前などの個人に関する情報の秘密は守られます。詳しくは以下の項目でご説明します。

### 11-1．あなたから得たデータがあなたのものだと特定されることはありません。

あなたの研究データはこの研究の目的（2-1．この臨床研究の目的参照）のために収集され、研究関係者（この病院の職員など）に利用されます。研究データを収集する際はコード化されるため、あなたのお名前や住所等の個人情報が集められることはなく、報告書からそのデータがあなたのものであると特定されることはありません。

### 11-2．研究中あるいは研究終了後に、カルテなどからあなたの情報が調査されることがありますが、あなたに関する情報の秘密は守られます。

法令や指針等に従って適切に研究が行われているか、データの品質に問題がないかなどを確認するために、モニタリングや監査が行われます。モニタリングや監査の委託を受けたこの病院や企業等の職員、または厚生労働省や臨床研究を審査する委員会のメンバーが、あなたのカルテなどの医療記録を見ることがあります。しかし、これらの人には守秘義務があるため、あなたに関する情報の秘密は守られます。

### 11-3．この研究結果が公表される場合でも、あなたの身元が明らかになることはありません

この研究で得られた成績は、医学雑誌などに公表されことがあります。またこの機器が保険診療で使えるようになるために、厚生労働省にデータを提出することがあります。いずれの場合も、研究データとして収集されたデータのみが解析され、

公表されます。お名前や住所等の個人情報は一切公表されません。このため、あなたのプライバシーは守られます。

## 12. この臨床研究に関する情報公開の方法

この臨床研究に関する情報は、厚生労働省が整備するデータベース Japan Registry of Clinical Trials（jRCT：臨床研究等提出・公開システム）に登録し、情報公開されています。この臨床研究が終了次第、研究の結果についても、jRCT に公開されます。

また、この研究の結果が公表される場合にも、あなたの名前などの個人情報は一切わからないようにします。（URL：https://jrct.niph.go.jp/search）

## 13. この臨床研究の実施に関する資料の開示、閲覧について

あなたのデータがどのような形で提供され、どのように利用されるかを確認したい場合は、担当の医師にご相談下さい。研究計画書、同意説明文書を閲覧することが可能です。しかし、研究上の機密に関わる内容が含まれる場合、要求される全ての情報開示に応じることはできませんのでご了承下さい。

## 14. データ等の保管および廃棄の方法と二次利用について

### 14-1. データ等の保管および廃棄の方法

研究のために集められたデータは、適切に保管されます。診療録などは医療機関で保管されますが、個人を特定できるような記載を削除した情報や研究に関わる重要な文書などは、研究責任医師が保管します。保管する期間は、研究終了後５年を経過した日までとなりますが、研究の公正性を保つために、それ以上長期で保管する場合もあります。保管期間終了後、紙媒体で保管されていたデータは、シュレッダーなどで再現不可能な状態にしたうえで廃棄します。

### 14-2. データ等の二次利用について

あなたにはこの研究の参加に加えて、将来の研究での使用を目的としたデータ等の提供および保管と研究への使用の可否について、お考えいただきたいと思いま

す。具体的な研究内容はまだ決まっていませんが、将来、この研究の関係者で検討して決定された後、法令等に従い倫理委員会に申請し承認を得た上で実施されます。データ等は、最終的な研究の結果が報告されてから最長で５年安全に保管され、その後廃棄されます。

## 15. 知的財産権と利益相反について

本研究の結果が特許権等の知的財産を生み出す可能性がありますが、その場合の知的財産権は研究者もしくは所属する研究機関に帰属します。

本研究の実施に関して、利益相反（起こりうる利害の衝突）が存在しないことを確認しています。

本研究は、令和４年度成長型中小企業等研究開発支援事業(Go-Tech 事業)の研究助成を得て実施します。研究資金については、資金計画に基づいて運用され、研究責任医師によって適切に管理されます。

株式会社Ｐ・マインドが、本研究の試験機器および機器に関する情報の提供を行い、研究資金の提供を行います。なお、本研究は当院の臨床研究利益相反管理委員会の審議を受け、適切に利益相反の管理が行われています。また、千葉大学臨床研究審査委員会の審査を受けています。

利益相反（Conflict of Interest: COI）とは、企業の研究への関与や、研究に関わる企業と研究者との間に経済的利益関係が存在することにより、公正かつ適正な判断が損なわれると第３者から懸念されかねない状態のことです。これにより、研究の信頼性が損なわれ、患者さんの保護がおろそかになる可能性があります。一方、臨床研究を適切に実施するためには一定の研究資金の確保や物品の提供を受けることは必要であり、研究者が企業からこれらの援助を受けること自体に問題はありません。このため、起こりうる利益相反を適切に管理し、十分に説明を行うことにより臨床研究に対する信頼を得ることが必要です。

## 16. あなたの費用負担について、この臨床研究に参加中の負担を軽減するための費用について

本研究で使用される試験機器および検査の費用は、研究費によってまかなわれます。原疾患および他の合併症に対する検査・処置・治療が通常の保険診療にて行われます。そのため、通常の診療と同じように費用のご負担がありますが、研究の参加に伴って特別に費用のご負担が増えることはありません。

＜負担軽減費の支払いがある場合は以下のテンプレートをご使用ください＞

この試験に参加するための通院に要する交通費や患者さんの負担を軽減するための費用として、「負担軽減費」を研究費からお支払いします。スクリーニング、Day1、４週、８週、12 週、16 週、20 週などの最大７回の来院について、１回の来院あたり、7000 円をお支払いいたします。

＜負担軽減費を支払わない場合は以下のテンプレートをご使用ください＞

この試験に参加するにあたって、あなたに支払われる費用はありません。

## 17. 臨床研究の審査について（臨床研究審査委員会について）

千葉大学では学長が医学部附属病院内に臨床研究審査委員会を設置しており、医学または医療の専門家、臨床研究の対象者の保護および医学または医療分野における人権の尊重に関して理解のある法律に関する専門家または生命倫理に関する識見を有する方や、上記に掲げる者以外の一般の立場の方に委員となっていただき、倫理的および科学的観点から、臨床研究の実施内容に問題がないかどうかを審査しています。

臨床研究審査委員会の名称：千葉大学　臨床研究審査委員会
臨床研究審査委員会の設置者：千葉大学　学長
臨床研究審査委員会の所在地：千葉県千葉市中央区亥鼻 1-8-1
URL：https://jcrb.niph.go.jp/applications/detail/55

## 18. 研究組織について

この研究は以下の医療機関にて実施する、多施設共同研究です。

【千葉大学医学部附属病院】

研究代表（責任）医師：甲賀　かをり

【東京大学医学部附属病院】

研究責任医師：泉　玄太郎

【山梨大学医学部附属病院】

研究責任医師：吉野　修

【福岡大学病院】

研究責任医師：四元　房典

【よこすか内科小児科・はるこレディースクリニック】

研究責任医師：横須賀　治子

【聖順会ジュノ・ヴェスタクリニック八田】

研究責任医師：八田　真理子

【医療法人ヒューマンリプロダクション　つばきウイメンズクリニック】

研究責任医師：鍋田　基生

【医療法人かしわ会　かしわざき産婦人科】

研究責任医師：柏崎　祐士

## 19．研究担当医師の連絡先および相談窓口（苦情および問い合わせ）

あなたがこの研究について知りたいことや、心配なことがありましたら、遠慮なく担当医師または患者相談窓口にご相談下さい。

＜千葉大学医学部附属病院以外の施設においては各施設にて以下のテンプレートを使用し、千葉大学医学部附属病院の問い合わせ先を削除する。＞

〇〇病院

研究責任医師：〇〇〇科　〇〇〇〇　　電話番号〇〇-〇〇-〇〇（内線〇〇）

研究担当医師：〇〇〇科　〇〇〇〇　　電話番号〇〇-〇〇-〇〇（内線〇〇）

患者相談窓口

月～金（9:00～17:00）：電話番号〇〇-〇〇-〇〇（内線〇〇）

緊急時夜間・休日相談窓口：電話番号〇〇-〇〇-〇〇（内線〇〇）

夜間・休日連絡先は、体調が優れず、急遽連絡が必要な場合にご利用ください。

〇〇科の臨床研究に参加していることをお伝えください。

＜千葉大学医学部附属病院では以下の問い合わせ先を記載し、上記の他施設の問い合わせ先を削除する。＞

千葉大学医学部附属病院（代表電話 043-222-7171）

研究責任医師：婦人科　甲賀かをり　（内線：5314）

研究担当医師：婦人科　石川博士　（内線：5314）

患者相談窓口

月～金（9:00～17:00）：電話番号 043-222-7171（内線：6090）

臨床試験部

月～金（8:30～17:00）：電話番号 043-222-7171（内線：6460）

緊急時夜間・休日相談窓口：代表電話（043-222-7171）

夜間・休日連絡先は、体調が優れず急遽連絡が必要な場合にご利用ください。

婦人科の臨床研究に参加していることお伝えください。

医師

# 同 意 文 書

臨床研究課題名：子宮内膜症に対するAT-04の有効性および安全性に関するシャム機対照二重盲検並行群間比較試験

＜説明事項＞

1. 臨床研究とは？
2. この臨床研究について
3. 臨床研究の方法と期間
4. この臨床研究の実施により予期される利益と不利益について
5. この研究に参加しない場合の他の治療方法と予期される利益と不利益について
6. あなたの健康に被害が生じた場合について
7. この臨床研究への参加は患者さんの自由な意思によるものです。
8. この臨床研究に関する情報は、随時ご連絡します
9. この臨床研究を中止させていただく場合があります
10. この臨床研究への参加に同意された場合は、次の点を守って下さい
11. あなたの個人情報の取り扱いについて
12. この臨床研究に関する情報公開の方法
13. この臨床研究の実施に関する資料の開示、閲覧について
14. データ等の保管および廃棄の方法と二次利用について
15. 知的財産権と利益相反について
16. あなたの費用負担について、この臨床研究に参加中の負担を軽減するための費用について
17. 臨床研究の審査について（臨床研究審査委員会について）
18. 研究組織について
19. 研究担当医師の連絡先および相談窓口（苦情および問い合わせ）

【患者さんの署名欄】

私はこの研究に参加するにあたり、上記の事項について十分な説明を受け、同意説明文書を受け取り、内容等を十分理解いたしましたので、この研究に参加することに同意します。

同意日：　　年　　月　　日

患者氏名：＿＿＿＿＿＿＿＿＿＿　（自署）

【医師の署名欄】

私は、上記患者さんに、この研究について十分に説明いたしました。

説明日：　　年　　月　　日

所属：

氏名：＿＿＿＿＿＿＿＿＿＿　（自署）

患者用

# 同 意 文 書

臨床研究課題名：子宮内膜症に対するAT-04の有効性および安全性に関するシャム機対照二重盲検並行群間比較試験

＜説明事項＞

1. 臨床研究とは？
2. この臨床研究について
3. 臨床研究の方法と期間
4. この臨床研究の実施により予期される利益と不利益について
5. この研究に参加しない場合の他の治療方法と予期される利益と不利益について
6. あなたの健康に被害が生じた場合について
7. この臨床研究への参加は患者さんの自由な意思によるものです。
8. この臨床研究に関する情報は、随時ご連絡します
9. この臨床研究を中止させていただく場合があります
10. この臨床研究への参加に同意された場合は、次の点を守って下さい
11. あなたの個人情報の取り扱いについて
12. この臨床研究に関する情報公開の方法
13. この臨床研究の実施に関する資料の開示、閲覧について
14. データ等の保管および廃棄の方法と二次利用について
15. 知的財産権と利益相反について
16. あなたの費用負担について、この臨床研究に参加中の負担を軽減するための費用について
17. 臨床研究の審査について（臨床研究審査委員会について）
18. 研究組織について
19. 研究担当医師の連絡先および相談窓口（苦情および問い合わせ）

【患者さんの署名欄】

私はこの研究に参加するにあたり、上記の事項について十分な説明を受け、同意説明文書を受け取り、内容等を十分理解いたしましたので、この研究に参加することに同意します。

同意日：　　　年　　　月　　　日

患者氏名：＿＿＿＿＿＿＿＿＿＿＿＿　（自署）

【医師の署名欄】

私は、上記患者さんに、この研究について十分に説明いたしました。

説明日：　　　年　　月　　日

所属：

氏名：＿＿＿＿＿＿＿＿＿＿＿＿　（自署）

事務局

# 同 意 文 書

臨床研究課題名：子宮内膜症に対するAT-04の有効性および安全性に関するシャム機対照二重盲検並行群間比較試験

<説明事項>

1. 臨床研究とは？
2. この臨床研究について
3. 臨床研究の方法と期間
4. この臨床研究の実施により予期される利益と不利益について
5. この研究に参加しない場合の他の治療方法と予期される利益と不利益について
6. あなたの健康に被害が生じた場合について
7. この臨床研究への参加は患者さんの自由な意思によるものです。
8. この臨床研究に関する情報は、随時ご連絡します
9. この臨床研究を中止させていただく場合があります
10. この臨床研究への参加に同意された場合は、次の点を守って下さい
11. あなたの個人情報の取り扱いについて
12. この臨床研究に関する情報公開の方法
13. この臨床研究の実施に関する資料の開示、閲覧について
14. データ等の保管および廃棄の方法と二次利用について
15. 知的財産権と利益相反について
16. あなたの費用負担について、この臨床研究に参加中の負担を軽減するための費用について
17. 臨床研究の審査について（臨床研究審査委員会について）
18. 研究組織について
19. 研究担当医師の連絡先および相談窓口（苦情および問い合わせ）

【患者さんの署名欄】

私はこの研究に参加するにあたり、上記の事項について十分な説明を受け、同意説明文書を受け取り、内容等を十分理解いたしましたので、この研究に参加することに同意します。

同意日：　　年　　月　　日

患者氏名：＿＿＿＿＿＿＿＿＿＿＿＿　（自署）

【医師の署名欄】

私は、上記患者さんに、この研究について十分に説明いたしました。

説明日：　　年　　月　　日

所属：

氏名：＿＿＿＿＿＿＿＿＿＿＿＿　（自署）
